# The Efficacy of MDMA (3,4-Methylenedioxymethamphetamine) for Post-traumatic Stress Disorder in Humans: A Systematic Review and Meta-Analysis

**DOI:** 10.7759/cureus.15070

**Published:** 2021-05-17

**Authors:** Sarah Tedesco, Ganeya Gajaram, Shahzad Chida, Arham Ahmad, Meghan Pentak, Marina Kelada, Layth Lewis, Deepa Krishnan, Carolyn Tran, Oladipo T Soetan, Lawrance T Mukona, Ayodeji Jolayemi

**Affiliations:** 1 Psychiatry, American University of Antigua College of Medicine - Interfaith Medical Center, Brooklyn, USA; 2 Psychiatry, Interfaith Medical Center, Brooklyn, USA; 3 Internal Medicine, Richmond University Medical Center, Staten Island, USA; 4 Psychiatry, American University of Antigua, New York, USA; 5 Psychiatry, Medical University of the Americas - Interfaith Medical Center, Brooklyn, USA; 6 Internal Medicine, American University of Antigua, Norwalk, USA

**Keywords:** posttraumatic stress, ptsd diagnosis and treatment, ptsd, mdma-assisted psychotherapy, mdma

## Abstract

*Background*: 3,4-methylenedioxymethamphetamine (MDMA), known recreationally as “Molly” or “Ecstasy”, is a triple monoamine reuptake inhibitor. MDMA specifically acts as a weak 5-HT1 and 5-HT2 receptor agonist, targeting 5-HT_2A_, 5-HT_2B_, and 5-HT_2C _receptors. Its potential use for therapeutic purposes with these pharmacological profiles remains a controversial subject. Studies have shown the potential benefits in clinical trials for post-traumatic stress disorder (PTSD). A larger amount of data has been provided for the push in support of MDMA-assisted psychotherapy in these patients.

*Objective*: The aim of this article is to compute a meta-analysis and conduct a systematic review of the effects of MDMA on PTSD, discussing the potential benefits and adverse events relative to dosing and stability of treatment.

*Methods*: Articles were collected and analyzed for systematic review: 16 articles were included in the systematic review that met the criteria for the use of MDMA in the treatment of PTSD as well as assessing the safety and efficacy of the drug in human participants. Ten studies were used for the meta-analysis, with a cumulative sample size of 168 patients. The significance of the findings on dosing and efficacy of MDMA in healthy human participants was quantified based on the Clinician-Administered PTSD Scale for DSM-5 (CAPS-5) and PTSD symptom scores.

*Results*: The disorders for which MDMA demonstrated a net positive or net negative effect on symptoms are presented separately. Adverse events in patients across all disease classes are presented. The therapeutic index for patients who demonstrated a benefit is also presented. An odds ratio for beneficial and adverse events is used to determine treatment-resistant patients who may benefit from clinical trials of MDMA.

*Discussion*: Findings show promising evidence for the potential therapeutic use of MDMA alongside psychotherapy in the treatment of PTSD. The pharmacological profile of MDMA may provide direction for future drug developments to treat patients with treatment-resistant psychiatric disorders.

## Introduction and background

3,4-methylenedioxymethamphetamine (MDMA), known recreationally as “Molly” or “Ecstasy”, is a triple monoamine reuptake inhibitor that acts as a stimulant and hallucinogen [1]. It is a recreational drug that produces an energizing effect, distortions in time and perception, and enhanced enjoyment from sensory experiences [1]. Years of reported experiences by recreational users combined with research data collected over the last 50 years have allowed the once “dangerous and messy drug” to be approved by the United States Food and Drug Administration (FDA) for its use in psychiatric treatment [2]. Studies on psychiatric treatments for mental illness have shown that there is a general dose-dependent benefit of MDMA in assisted psychotherapy, where the Clinician-Administered PTSD Scale (CAPS) total scores at the primary endpoint showed changes proportional to the dosage amount [3]. There are, however, adverse health effects with recreational use due to overdosing, such as hypertension, faintness, panic attacks, and, in severe cases, loss of consciousness and seizures [4]. There is still much to be learned about MDMA’s therapeutic dosing effect and its support with psychotherapy treatment of PTSD.

Post-traumatic Stress Disorder (PTSD) is a complex mental illness affecting 7.7 million adults in the United States every year, with 1 in 11 people diagnosed with PTSD in their lifetime [2]. PTSD affects more than twice as many women (10%) as men (4%) with sexual assault being the leading traumatic event [5]. As per the diagnostic criteria in the fifth edition of the Diagnostic and Statistical Manual of Mental Disorders (DSM-5), PTSD is included as a new category in DSM-5, Trauma and Stressor-Related Disorders [5]. All the conditions included in this classification require exposure to a traumatic or stressful event as a diagnostic criterion. At least one criterion in each of the following categories must be met in order to validate a PTSD diagnosis: exposure to death, threatened death, serious injury or actual/threatened sexual violence; persistent re-experience of the traumatic event; avoidance behaviors; negative alterations in cognitions and mood; vivid trauma-related arousal and reactivity that began or worsened after the trauma, and functional significance with symptoms lasting for more than 1 month [5].

The mainstay treatment for PTSD has traditionally consisted of exposure-based therapies directed at the revisualization of the patient’s initial traumatic experiences [6]. When patients are exposed to occurrences that trigger traumatic events while under the therapeutic treatment, this enables them to reactivate their fear response and work on separating these traumatic triggers from the conditioned response [4]. MDMA-assisted psychotherapy theoretically targets both the memory reconsolidation and fear extinction processes [4]. The term “memory reconsolidation” describes a type of neuroplasticity that involves the process of an established memory being reactivated, destabilized, and then modified or updated with additional information [4]. Hypothetically, when trauma memories are retrieved while under the influence of MDMA during therapy, a strong prediction error is generated by the unique internal state of MDMA-stimulated elevation of neurochemicals, hormones, and the supportive therapeutic setting [4]. This mismatch of experiences, such as recall of memory with strong fear/anxiety versus recall with emotions such as love or empathy, would allow for an update of the information through molecular mechanisms [4]. MDMA increases the release of dopamine (DA) in the striatum and midbrain. Dopamine positively correlates with prediction error, and therefore, MDMA-stimulated DA efflux may amplify and drive a prediction error related to the traumatic memory [4].

Clinicians have found that MDMA can be purified and used to facilitate the above-mentioned therapeutic effects [6]. MDMA promotes the release of dopamine, serotonin, and norepinephrine in the mesolimbocortical circuitry of the brain, as well as the neurohormonal signaling of oxytocin, cortisol, prolactin, and vasopressin [7]. The comprehensive effect of these neurochemicals has been shown to enhance therapeutic success as well as decrease nonresponse and dropout rates [7]. Long-lasting PTSD remission and a reduction in symptoms have been reported after only two to three MDMA-assisted therapy sessions [7]. MDMA specifically acts as a weak 5-HT1 and 5-HT2 receptor agonist, targeting 5-HT_2A_, 5-HT_2B_, and 5-HT_2C_ receptors. 5-HT_2A_ affects neural activity, perception, cognition, and mood, playing a role in the regulation of behavior, including responses to anxiogenic situations and psychoactive substances. Studies have shown that MDMA-induced emotional excitability and positive mood are linked to the actions of 5-HT_2A_ receptors [8]. 5-HT_2B_ may play a role in the perception of pain and in the regulation of behavior, including impulsive behavior. 5-HT_2C_ regulates neuronal activity via the activation of short transient receptor potential calcium channels in the brain, and thereby modulates the activation of proopiomelanocortin neurons and the release of cortisol releasing hormone, which then regulates the release of corticosterone. 5-HT_2C_ has also demonstrated playing a role in responses to anxiogenic stimuli or stress, and in the regulation of appetite and eating behavior. This can be understood by the appetite-suppressing effects of MDMA use [8]. The pharmacokinetics of MDMA in humans has been characterized using oral doses of up to 150 mg [9]. MDMA disposition in the body demonstrates nonlinear pharmacokinetics. Metabolism of the drug has shown to result in N-demethylation to 3,4-methylenedioxyamphetamine (MDA). The MDMA metabolite MDA is a potent 5-HT_2B_ agonist which could contribute to a change in the perception of pain and in the regulation of behavior, including impulsive behavior [10]. It also undergoes further demethylation and its metabolites are known to be excreted in the urine as conjugated glucuronide or sulfate metabolites [9]. 

A commonly observed neurological feature of PTSD is the reduction of hippocampal volume [3]. The hippocampus is involved in the control of stress responses, declarative memory, and contextual aspects of fear conditioning [3]. Magnetic resonance imaging (MRI) studies demonstrated smaller hippocampal volumes in patients with PTSD compared with healthy controls [3]. MDMA has been shown to acutely decrease activity in the left amygdala and increase blood flow to the prefrontal cortex in the brain [11-13]. 

Diving deeper into the neurochemical features of PTSD, there is an array of abnormal chemical regulations to be found. These tend to include serotonin, dopamine, (nor)epinephrine, acetylcholine, glutamate brain-derived neurotrophic factor (BDNF), oxytocin, and cortisol, all of which are found in brain circuits that regulate and collaboratively integrate stress and fear responses [3]. While MDMA releases serotonin, norepinephrine, and dopamine in the brain, it also indirectly increases levels of the neurohormones oxytocin, arginine vasopressin, and cortisol in humans. The combined neurobiological effects of MDMA increase compassion and reduces defenses and fear of emotional injury while enhancing communication and introspection. MDMA also releases other downstream signaling molecules (BDNF) to dynamically modulate emotion memory circuits [4]. By reducing activation in brain regions implicated in the expression of fear-related behaviors and increasing connectivity between the amygdala and hippocampus, MDMA may allow for the reprocessing of traumatic memories and increase emotional engagement with therapeutic processes, thereby assisting with the psychotherapeutic treatment of PTSD [4].

A number of human studies have been able to explore such possible impacts of MDMA on PTSD, as well as document adverse events. This paper is a systematic literature review and meta-analysis of MDMA-assisted psychotherapy in which MDMA was administered to adults (mean age 35.7 to 47 years old) with CAPS scores of 50 or higher to assist in their psychotherapy treatment for PTSD. Primary measures such as CAPS scores, as well as secondary measures, were analyzed immediately after each trial and during several long-term follow-up (LTFU) periods. This article will build upon a recent meta-analysis by attempting to address some of its limitations, including a small sample size and limited generalizability of their results [6]. This article will aim to discuss the small window of optimal therapeutic threshold and dosage that MDMA will have in assisted psychotherapy for patients suffering from PTSD. We aim to expand upon the ten-study sample size in the aforementioned meta-analysis by presenting a larger sample size and further exploring the potential benefits and adverse events relative to MDMA-assisted psychotherapy for patients with PTSD.

## Review

Materials and methods

Search Strategy

**Figure 1:**
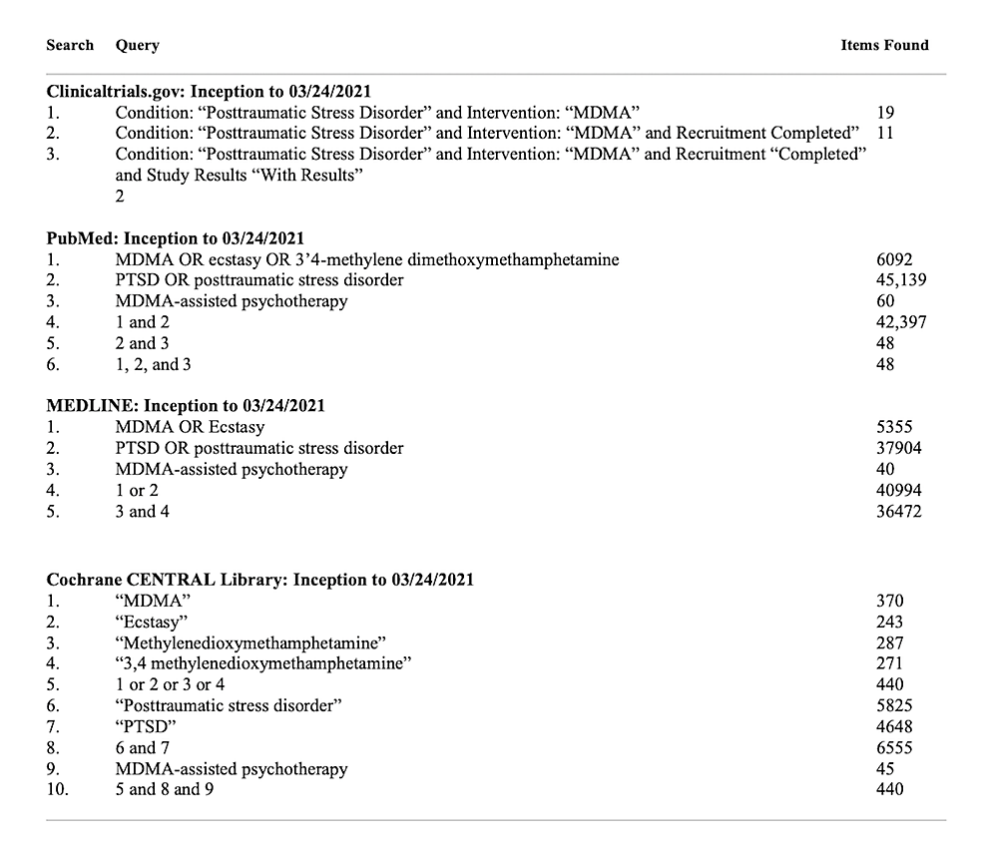
Systematic Review Protocol MDMA: 3,4-methylenedioxymethamphetamine; PTSD: post-traumatic stress disorder

**Figure 2:**
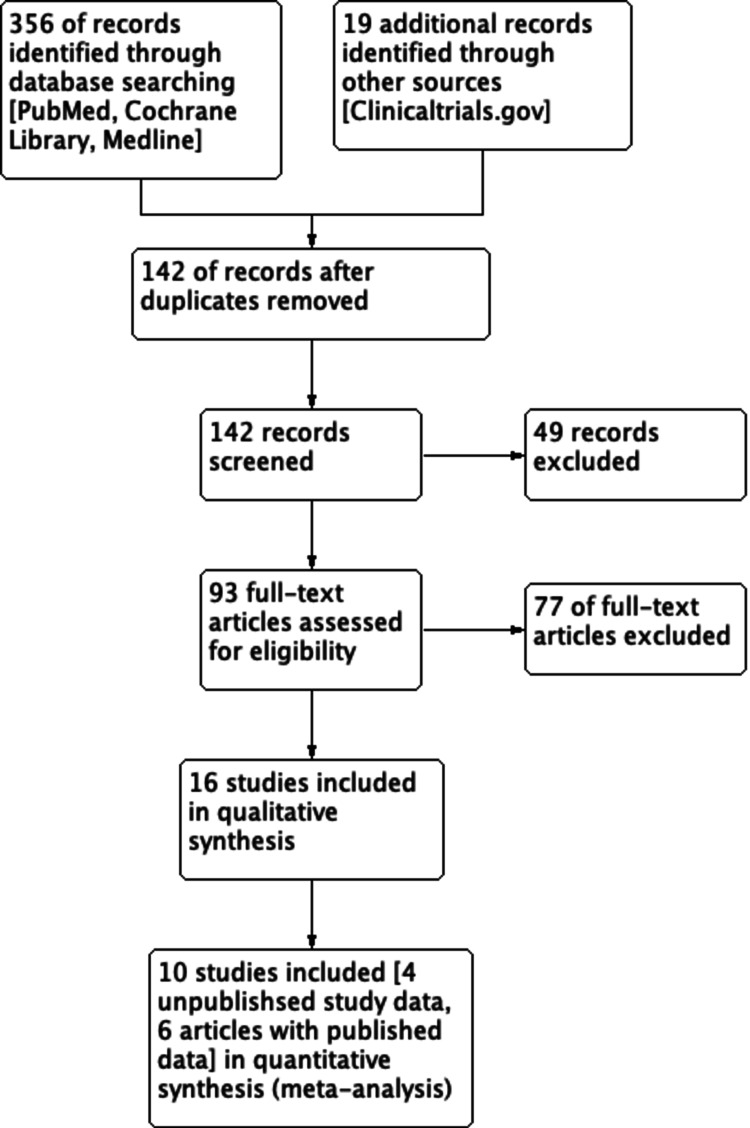
PRISMA Study Flow Diagram PRISMA: Preferred Reporting Items for Systematic Reviews and Meta-Analyses

This systematic review was performed in accordance with the PRISMA (Preferred Reporting Items for Systematic Reviews and Meta-Analyses) guidelines. We developed a search strategy to retrieve references relating to the treatment of PTSD using MDMA using the following keywords: “MDMA”, “MDMA assisted psychotherapy”, “ecstasy”, “3,4-methylenedioxymethamphetamine”, “posttraumatic stress disorder”, and “PTSD”. This strategy was replicated on each of the following databases: PubMed, Clinicaltrials.gov, MEDLINE, and Cochrane CENTRAL Library. Details of each strategy can be seen in Figure 1**.** Searches were conducted in February 2021 from inception in order to maximize yield and repeated on March 24, 2021 in order to prevent missing any recently published and completed articles. All searches included non-English language literature. No studies were found in languages other than English. Selected researchers were contacted who are active in the area seeking information about unpublished study reports. Reference lists of relevant review articles were manually searched to identify any further studies of interest not retrieved by electronic search. Figure 2 (PRISMA flow diagram)visualizes the search strategy for this systematic review and meta-analysis.

Eligibility Criteria

Studies included participants diagnosed with treatment-resistant PTSD per guidelines in DSM-5, CAPS, and PTSD symptom scales. Included studies had to administer MDMA in a controlled setting, or MDMA in combination with psychotherapy, with the aim of reducing the symptoms and signs of PTSD. Comparison interventions involved the use of different approaches including pharmacotherapy, placebo, and psychotherapy. The majority of the MDMA studies were administered by the Multidisciplinary Association for Psychedelic Studies (MAPS), which did allow for some concurrent psychiatric disorders with PTSD being the primary diagnosis. Studies that were conducted in both inpatient and outpatient trials were included. Randomized, quasi-randomized, and uncontrolled trials which provided detailed information on the MDMA dosage utilized and characteristics of participants treated were considered eligible. 

Study Selection and Data Extraction

Study selection and data extraction took place in three stages. During the first stage, four reviewers assessed 356 abstracts and article titles from the systematic search in correspondence to the inclusion and exclusion criteria. Agreed upon by all four reviewers, the inclusion criteria consisted of: patients with PTSD (men and non-pregnant women 18 years and older), prior pharmaceutical treatment for PTSD, duration of PTSD >6 months, prior psychotherapy, no history of drug abuse, and no history of another primary psychiatric diagnosis. Authors were not blinded from the names of journals, authors, institutions, and study results during study selection. All articles deemed relevant by any of the reviewers were included, given they did not meet the exclusion criteria. A total of 142 articles were identified during this stage, after the exclusion of all duplicate articles. Any pertinent duplicates were set aside for review at a later stage.

During the second stage of review and data extraction, all 10 authors independently assessed the full text of selected articles. Articles were further excluded if they met any of the exclusion criteria from the first stage (i.e., they were not randomized trials or did not report response to MDMA or MDMA-assisted psychotherapy in a controlled setting). Any discrepancies between authors at this stage were resolved by consensus. Key information was extracted by all authors independently using a data collection form to record information against the outcome measures (response, severity of symptoms, adverse effects, completion of treatment, and implementation of follow-up treatment). Details pertaining to the study, demographics, and psychiatric history variables coded are presented in Appendices. The coded variables were set by the lead author and agreed upon by all authors. The articles that were used to pilot the variables were those selected for meta-analysis and systematic review. Disagreement was resolved by consensus, and the consensus ratings were used in the analysis. Key findings of studies were summarized descriptively in the first instance and the capacity for quantitative meta-analysis was considered. The methodology for data extraction was adapted from the study by Bahji et al. (2020) [6]. Significant findings were qualitatively summarized and considered for use in quantitative meta-analysis. 

In the final stage, 10 unique articles meeting the inclusion and exclusion criteria were sourced for the meta-analysis. Articles that were excluded and reserved during stage 1 due to duplication were reassessed at this point. Six were identified as duplicates of the 10 selected studies. These were reviewed for relevancy and were included for subjective analysis during the systematic review. Figure 2 (PRISMA flow diagram) depicts the process by which articles were dissected and selected for analysis and review. 

Data analysis

The following outcome measures were considered: (1) number of participants showing clinically significant response or remission at the end of treatment as determined by self-report, clinician judgment, or PTSD rating scales; (2) intensity of PTSD symptoms as determined by scores on PTSD rating scales, the need for additional as-needed (*pro re nata*, PRN) medications in addition to the experimental intervention, or overall assessments by clinicians and participants; (3) nature, incidence and frequency of adverse effects and whether the planned medication regime was modified in response to adverse effects; (4) completion of scheduled treatment; (5) number of participants engaged in further treatment following completion of the trial. Treatment response criteria for each study are summarized in tables. Briefly, ‘response’ was qualified by the percentage of participants no longer meeting PTSD diagnostic criteria using CAPS [14] or using minimum score thresholds for improvement. CAPS scores were equalized using a CAPS-4 conversion formula to CAPS-5 for more accurate and precise data. Two studies involved three treatment arms representing three different doses of MDMA [3,10]. The active medications, compared to each other, were included in separate subgroups and the calculation of overall totals was suppressed thereby avoiding the unit of analysis error of double-counting participants. 

Meta-analyses were performed with Review Manager 5.4 [15] using random-effects modeling. The effectiveness of MDMA-assisted psychotherapy was quantified using four measures: the likelihood of response indicated by the risk ratio (RR) comparing the proportion of participants with a clinically significant response between MDMA and control groups; the likelihood of remission indicated by the risk ratio (RR) comparing the proportion of participants with clinically significant remission between MDMA and control groups (which was operationalized using the loss of PTSD diagnostic status as a surrogate measure of remission); effect size, indicated by Standard Mean Difference (SMD) comparing the PTSD symptoms scores before and immediately after the intervention; and durability of the effect, the SMD comparing the PTSD symptoms scores before and in sustained follow-up sometime after treatment cessation.

Clinical and methodological heterogeneity was assessed by reviewing the variations between studies in terms of the characteristics of participants included, the dosage interventions, and documented outcomes. Quantitative heterogeneity was assessed statistically with the Chi^2^ test and its p-value, by visual inspection of the forest plots, the I^2^ statistic. A p-value of the Chi^2^ test lower than 0.05 or an I^2^ statistic of at least 50% indicated statistically significant heterogeneity. 

Studies were grouped for analysis by the nature of the outcome measure used. As there were considerable dosage values of MDMA used, subgroups, but not overall totals were calculated. The contribution of moderators to risk ratio (RR) or standard mean difference (SMD) variability across studies (either categorical or continuous) was further assessed in the systematic review as certain included studies had pooled data from the majority of the data collected in meta-analysis.

Assessment of Risk of Bias and Study Quality

**Figure 3:**
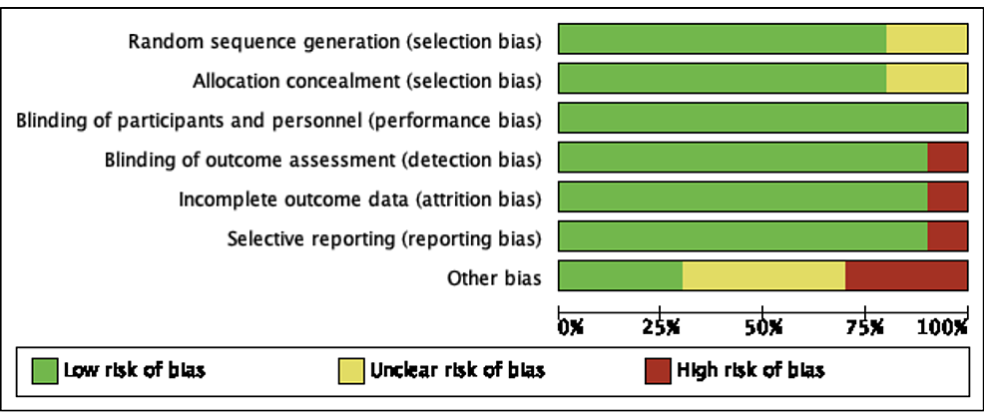
Risk of Bias Graph for All Included Studies [3,8,18-25]

**Figure 4:**
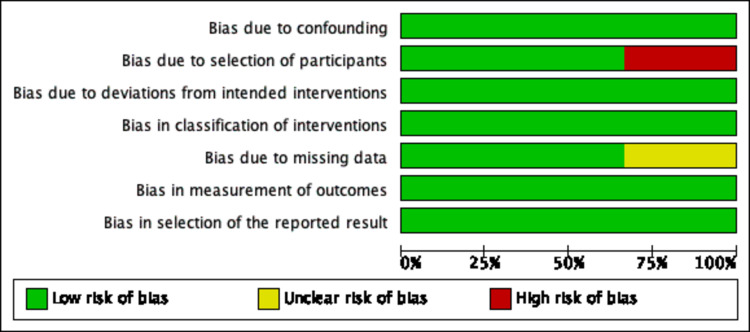
Risk of Bias Graph for Nonrandomized Trials MP-16, MP-17, MPVA-1 using ROBINS-I tool ROBINS-I: Risk Of Bias In Non-randomized Studies of Interventions [21-23]; MPVA-1 (Monson et al. 2019) [21]; MP-16: NCT03282123 [22]; MP-17: NCT03485287 [23]

**Figure 5:**
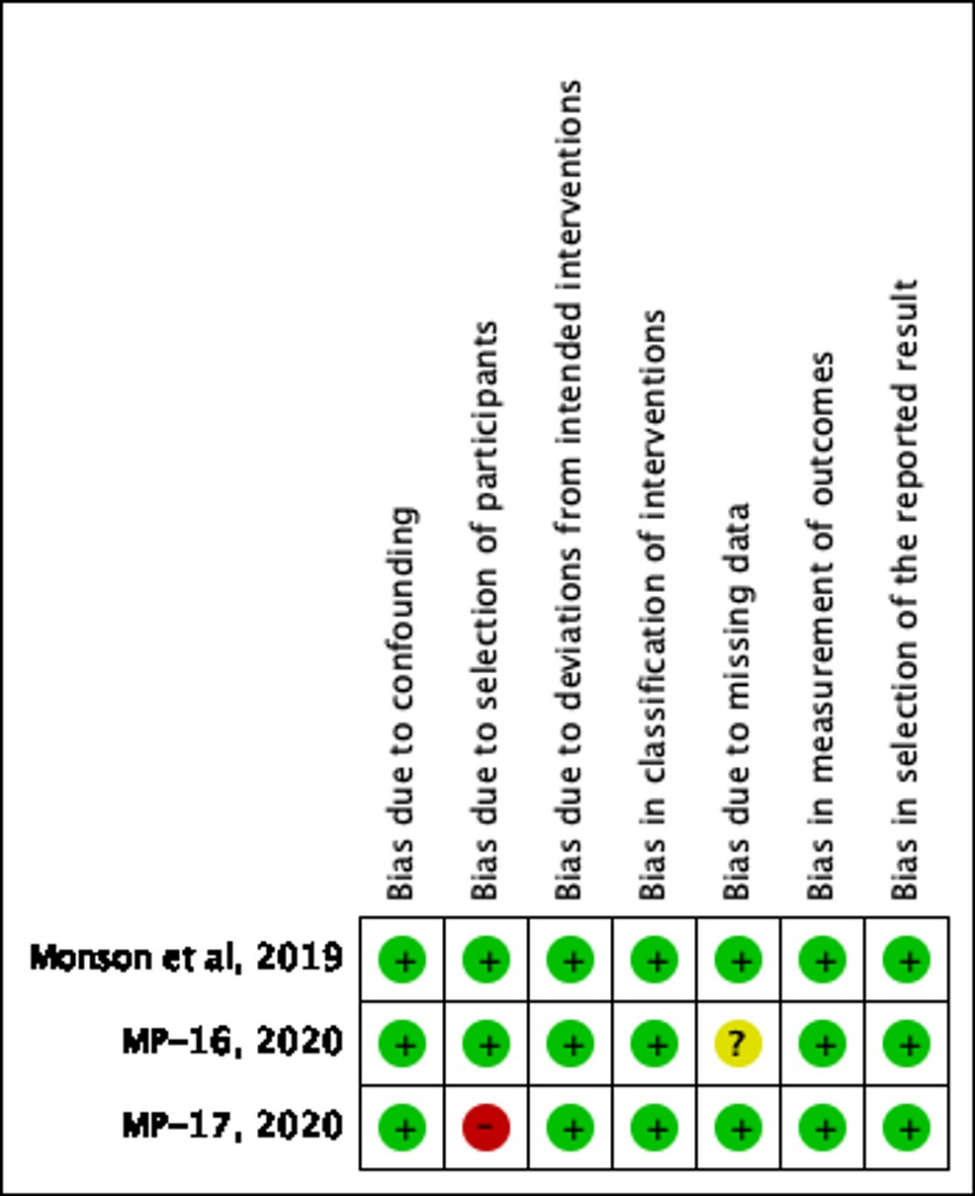
Risk of Bias Summary for Nonrandomized Trials MP-16, MP-17, MPVA-1 Using ROBT RevMan 5.4 ROBT: risk of bias tool; RevMan 5.4: Cochrane Review Manager version 5.4 MPVA-1 (Monson et al. 2019) [21]; MP-16: NCT03282123 [22]; MP-17: NCT03485287 [23]

**Figure 6:**
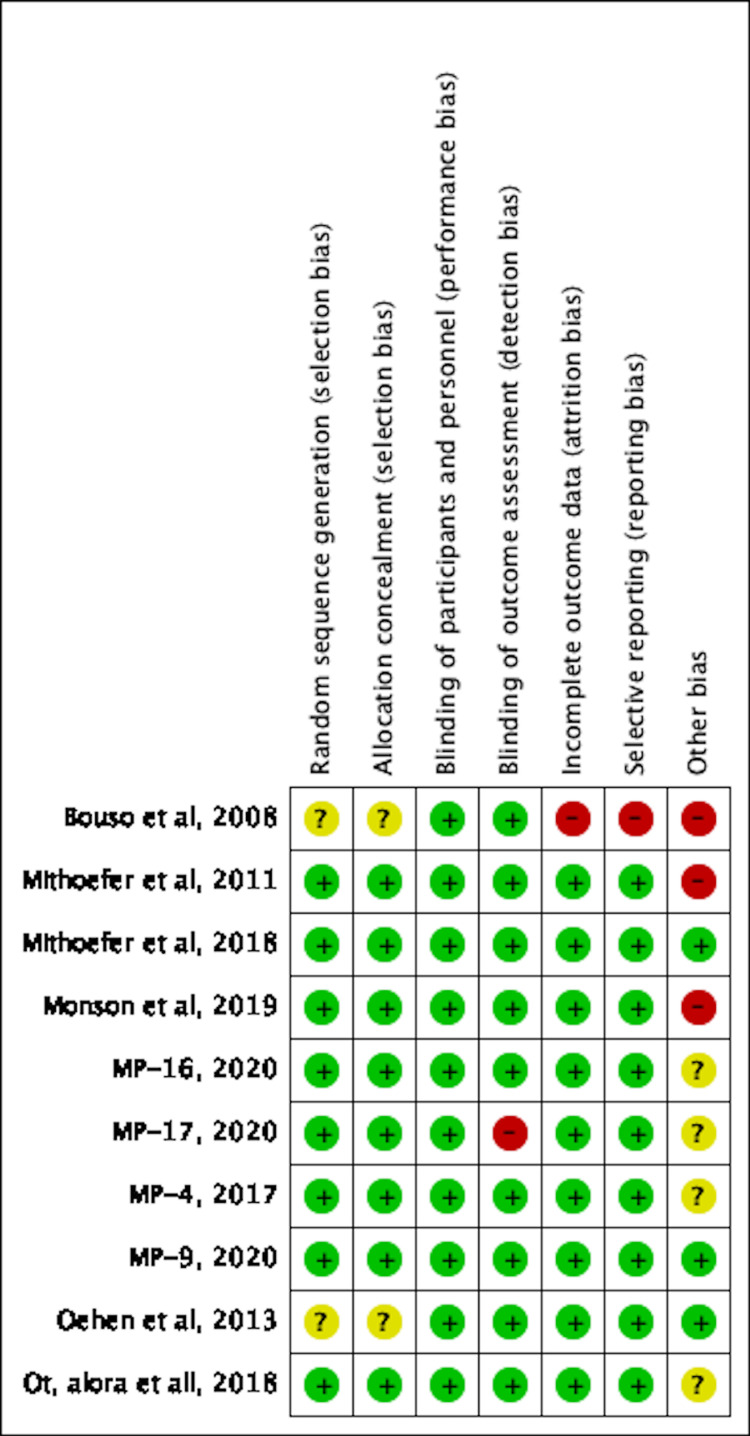
Risk of Bias Summary [3,8,18-25]; MP-16: NCT03282123 [22]; MP-17: NCT03485287 [23]; MP-4: NCT01958593 [24]; MP-9: NCT01689740 [25]

**Figure 7:**
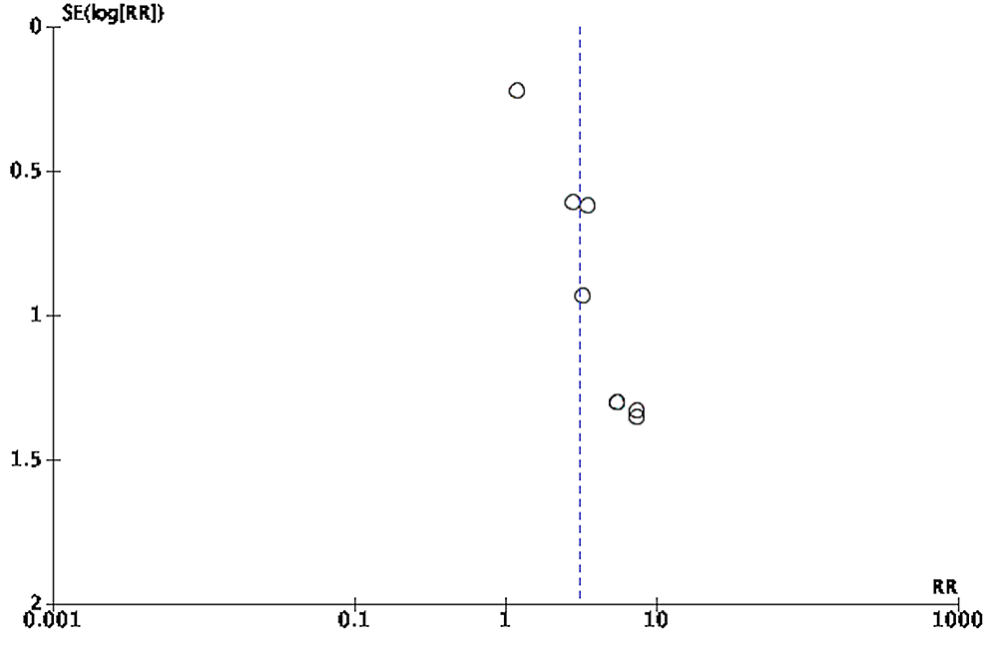
Funnel Plot of Risk Ratio of Responders to MDMA-Assisted Psychotherapy MDMA: 3,4-methylenedioxymethamphetamine [3,8,18-25]

**Figure 8:**
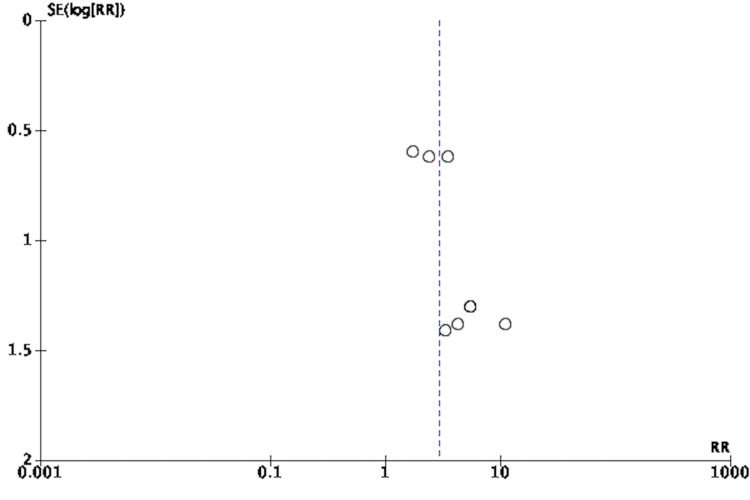
Funnel Plot of Standardized Mean Difference of Pre- Versus Post-Effect of MDMA-Assisted Psychotherapy MDMA: 3,4-methylenedioxymethamphetamine [3,8,18-25]

**Figure 9:**
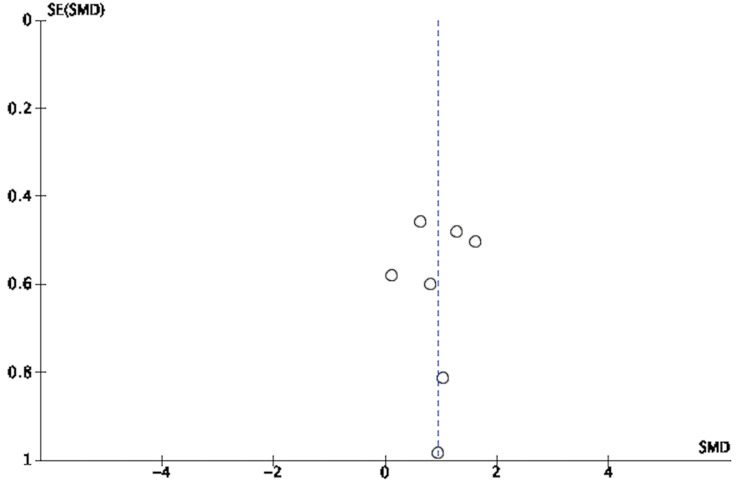
Funnel Plot of Standardized Mean Difference of Pre- Versus Follow-up Effect of MDMA-Assisted Psychotherapy MDMA: 3,4-methylenedioxymethamphetamine [3,8,18-25]

**Figure 10:**
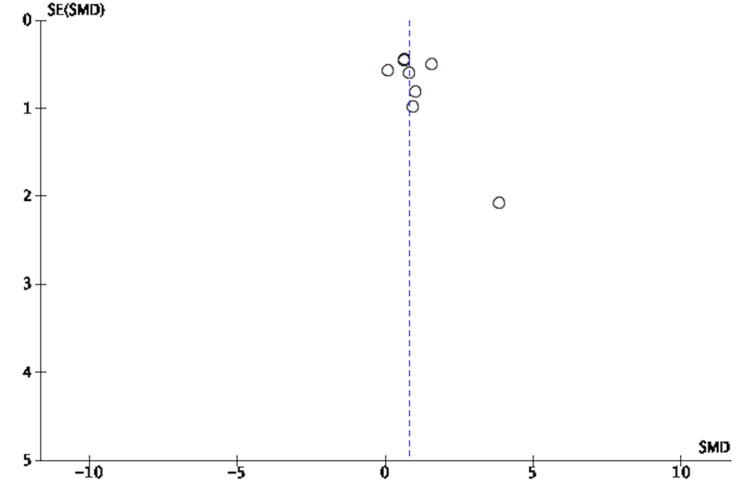
Funnel Plot for Those Who Went Into Remission [3,8,18-25]

An adequate amount of information was extracted based on included studied reports to enable assessment of the risk and bias. Study quality was assessed using the Cochrane Risk of Bias Tool (RoBT) for randomized, quasi-randomized controlled, and uncontrolled/open-label trials [15]. Study quality was assessed for uncontrolled non-randomized trials with the Cochrane RoBT with the Risk Of Bias In Non-randomized Studies of Interventions (ROBINS-I) assessment tool [16,17]. Refer to Figures 3-6. Four authors autonomously rated studies using the ROBINS-I tool and RoBT while the remaining authors verified independently: discrepancies were rectified based on consensus, and the consensus rating was used in analyses [16,17]. Publication bias was qualitatively evaluated by visually assessing the funnel plots for asymmetry, found in Figures 7-10.

Results

Systematic Review

Our search strategy identified 142 unique records, from which 93 different studies were isolated as potentially relevant to this review (Figure 2). Ten studies [3,8,18-25] with a total of 168 participants with PTSD were ultimately included in the meta-analysis. The individual original study characteristics have been summarized in Tables 1, 2. Mithoefer et al., 2018 [8] was a follow-up of the study Mithoefer et al., 2013 [26], and as a result, was not treated as a separate report. 

**Table 1:** Characteristics of Included Studies for Meta-Analysis Continued in Table 2 PTSD: post-traumatic stress disorder MP-16: NCT03282123 [22]; MP-17: NCT03485287 [23]; MP-4: NCT01958593 [24]; MP-9: NCT01689740 [25]

Study	NCT #	Country	N (M/F)	Age (mean, SD) [years]	Comorbid Depression n(%)	Comorbid Anxiety (n, %)	PTSD Duration (mean, SD) [months]	Prior Therapy (mean, SD) [months]	Primary Trauma type (%)
Bouso et al, 2008 [18]	n/a	Spain	6 (0/6)	35.7 (7.3)	0	0	265 (206)	>1 (n/a)	Sexual (100%)
Mithoefer et al, 2011 [19]	NCT00090064	USA	20 (3/17)	40.4 (7.2)	16 (80%)	3 (15%)	248 (173)	58.5 (49.5)	Crime/War (100%)
Oehen et al, 2011 [20]	NCT00343938	Switzerland	12 (2/10)	41.4 (11.2)	10 (83%)	1 (8%)	220 (144)	85.8 (71.4)	Sexual (58%)
Ot'alora et al, 2018 [3]	NCT01793610	USA	28 (19/9)	42.0 (12.9)	19 (68%)	15 (54%)	353 (232)	> 1 (n/a)	Occupational (100%)
Monson et al, 2019 [21]	NCT02876172	USA	12 (6/6)	47 (8)	10 (83%)	9 (72%)	at least 6 months did not specify the mean months duration	100% (all received prior psychotherapy)	childhood sexual abuse (50%); childhood physical abuse/neglect (33.3%); adult combat (16.7%)
Mithoefer et al, 2018 [8]	NCT01211405	USA	26 (19/7)	37.2 (10.3)	20 (77%)	11 (42%)	85.4 (63.9)	>1 (n/a)	occupational (100%)
MP-16 [22]	NCT03282123	USA	32 (male and female)	18 or older (n/a)	n/a	n/a	>6 months (n/a)	n/a	n/a
MP-17 [23]	NCT03485287	Canada	4 (male and female)	18 or older (n/a)	n/a	n/a	>6 months (n/a)	n/a	n/a
MP-4 [24]	NCT01958593	Canada	6 (male and female)	21 or older (n/a)	n/a	n/a	> 6 months (n/a)	n/a	n/a
MP-9 [25]	NCT01689740	Israel	10 (6/4)	18-65 (n/a)	n/a	n/a	>6 months (n/a)	n/a	n/a

**Table 2:** Characteristics of Included Studies for Meta-Analysis Continued from Table 1 MDMA: 3,4-methylenedioxymethamphetamine; CAPS: Clinician-Administered PTSD Scale; PTSD: post-traumatic stress disorder; SSSPTSD: Short Screening Scale for Post-traumatic Stress Disorder MP-16: NCT03282123 [22]; MP-17: NCT03485287 [23]; MP-4: NCT01958593 [24]; MP-9: NCT01689740 [25]

Study	PTSD scale used	Baseline Severity (mean, SD)	N (Experiment/Control)	MDMA Dosing	Number of MDMA Sessions	Nature of Control Group	Short-Term Follow-up	Long-Term Follow up Period	Response Criteria	Remission criteria	Adverse events
Bouso et al, 2008 [18]	SSSPTSD	41.5 (5.9)	4 (2)	50, 75 mg	1	Placebo	1-2 months	12 months	Operationalized	No longer meeting PTSD criteria	No drug-related serious adverse events occurred
Mithoefer et al, 2011 [19]	CAPS	79.4 (22.4)	12 (8)	125 mg	2	placebo	1-2 months	17-74 months	> 30% reduction in baseline CAPS score	No longer meeting PTSD criteria	No drug-related serious adverse events occurred
Oehen et al, 2011 [20]	CAPS	64.9 (15.7)	8 (4)	125 mg	3	25 mg MDMA	1-2 months	12 months	>15-point change in baseline CAPS score	No longer meeting PTSD criteria	No drug-related serious adverse events occurred
Ot'alora et al, 2018 [3]	CAPS	92.0 (18.0)	22 (6)	40, 75, 125 mg	3	40 mg MDMA	1-2 months	12 months	> 30% reduction in baseline CAPS	No longer meeting PTSD criteria	No drug-related serious adverse events occurred
Monson et al, 2019 [21]	CAPS-5	41.42 (5.76)	6 (6)	(75 mg, 100 mg) + optional half dose	2	uncontrolled; partner did not have PTSD criteria	3 months	6 months	>50% reduction in CAPS	No longer meeting PTSD criteria	no serious adverse events; diminished appetite, anxiety, headache and jaw tightness.
Mithoefer et al, 2018 [8]	CAPS-4	86.6 (28.2)	12 (14)	30, 75, 125 mg	3	30 mg MDMA	1-2 months	12 months	>30% reduction in baseline CAPS	No longer meeting PTSD criteria	1 possible drug-related serious adverse event (depression with suicidal ideation)
MP-16 [22]	CAPS-5	45.42 (6.70)	32 (0)	80 mg - 180 mg	3	none	4-5 months	n/a	31 point change in baseline CAPS score	no longer meeting PTSD criteria (24/32~75%)	no serious adverse events are noted
MP-17 [23]	CAPS-5	45.25 (11.84)	4 (0)	100 mg to 187.5 mg	3	none	4-5 months	n/a	>50 % reduction in baseline caps	no longer meeting PTSD criteria (all remissed)	no serious adverse events noted
MP-4 [24]	CAPS-4	87 (12.4)	4 (2)	75-125 mg	2	Placebo	0-3 months	12 months	>20% reduction in baseline caps	no longer meeting PTSD criteria	no serious adverse events noted
MP-9 [25]	CAPS-4	84.51 (12)	3 (7)	25 mg and 125 mg	2	Placebo	2 months	12 months	~30% reduction in baseline CAPS	no longer meeting PTSD criteria (2/5)	no serious adverse events noted.

All 10 studies were undertaken primarily in outpatient settings. In all studies, participants in the intervention group were offered MDMA-assisted psychotherapy. As all the 10 studies were funded by MAPS, they used the same format of psychotherapy: a manualized supportive therapy modality developed by MAPS - the specific details of this method of psychotherapy are described in the MAPS therapy manual [27], which is available online (https://maps.org/research/mdma) [28].

The studies occurred from 2004 to 2020 and took place in five different countries. The percentage of female participants ranged from 27-100%, with mean age ranging from 35.7 to 47 years old. In all studies, psychiatric diagnoses were established using the Diagnostic and Statistical Manual of Mental Disorders, Fourth Edition, Text-Revision (DSM-IV-TR) and Fifth Edition (DSM-5) criteria. Across five studies, the known symptomatic duration in participants was consistent with chronic PTSD (7-29 years). The other five studies [21-25] mention PTSD duration lasting greater than six months. It is important to note that PTSD severity measured at baseline was similar between the experimental and the control group participants across all of the studies. The severity was measured using CAPS-4 or CAPS-5 in nine out of the 10 studies, while Bouso et al. [18] used the Severity of Symptoms Scale for PTSD (SSSPTSD). Chronic PTSD was defined as having PTSD for at least six months and failing to show significant clinical response to at least one first-line treatment option, such as antidepressants (Selective Serotonin Reuptake Inhibitors [SSRIs]) or psychotherapy (Cognitive Behavioral Therapy [CBT], prolonged exposure therapy, eye movement desensitization, and reprocessing). In at least five of the studies [3,8,18-21] a majority of participants had comorbid depression or anxiety, but these studies excluded participants with more severe and persistent comorbid psychiatric disorders such as schizophrenia, bipolar disorder, and severe substance use disorders. 

In comparing the effect size of MDMA at treatment exit and follow-up, Cohen’s d effect size calculation was performed using CAPS-5 and PTSD symptom scales, shown in Table 3. For Bouso et al. [18], when looking at effect size from before treatment with MDMA to after completion, the effect size was 3.41. From pre-treatment to long-term follow-up, which was 12 months for the study, Cohen’s d was 3.83. For MP-16 (NCT03282123) [22] and MP-17 (NCT03485287) [23], the effect size from pre-MDMA to post-MDMA treatment was 1.58, and from pre-MDMA to long-term follow-up, it was 0.23. For Monson et al. [21]**, **the effect size from before treatment to after was 2.1, while for pre-MDMA to long-term follow-up at six months, Cohen’s d was 2.25. Finally, for Jerome et al [29], from which Cohen’s d was calculated using the other six studies, the pre-treatment to post-treatment effect size was 1.58, while from pre-treatment to long-term follow-up, it was 0.23 at 12 months.

**Table 3:** Effects of MDMA at treatment exit and follow up MDMA: 3,4-methylenedioxymethamphetamine

Study	Pre- to post-treatment	Pre-treatment to long-term follow-up
Bouso et al, 2008 [18]	3.41	3.83
Jerome et al, 2020 [29]	1.58	0.23
MP-16 and MP-17 [22,23]	3.23	n/a
Monson et al, 2019 [21]	2.1	2.25

Despite qualitative homogeneity, there was significant heterogeneity in the nature of the experimental designs across studies. This limited the nature of the experimental designs across studies thereby narrowing the extent of possible analysis. MDMA dosage varied from 25 mg to 187.5 mg (Table 2). At least three studies [3,10,29] allowed participants in the experimental groups to receive a supplemental dose of MDMA within 2-3 hours of the initial dose. These additional doses were given at half the original dose strength to boost the therapeutic effect. Regarding prior or concurrent therapy, at least two studies discontinued participant antidepressant therapy at least one month prior to initiating the course of MDMA-assisted psychotherapy [18,19]. Two other studies allowed medication to be used until at least the point of recruitment and did not provide any information on concurrent antidepressant use during the study [18,10]. Five studies did not provide any information about prior therapy (Table 1). The participants in Mithoefer et al. [19] received prior therapy for a mean duration of 58.5 months, while participants in Oehen et al. [20] received prior therapy for a mean duration of 85.8 months. All of the participants in Monson et al. [21] had received prior psychotherapy. Further information for the articles that were excluded from meta-analysis but included in the systematic review is summarized in Table 4. They were included due to their relevant study outcomes and significant findings to the original 10 studies. 

**Table 4:** Studies Included for Systematic Review Illustrating study outcomes and findings for effects of MDMA on PTSD MDMA: 3,4-methylenedioxymethamphetamine: PTSD: post-traumatic stress disorder; CAPS: Clinician-Administered PTSD Scale

Study	Study outcomes	Primary Findings
Jerome et al, 2020 [29]	Examining long-term change in PTSD symptoms and additional benefits/harms after MDMA-assisted psychotherapy for treatment of PTSD in 107 participants across six study sites.	Participants were randomized to either control group (inactive placebo; 25mg, 30 mg, or 40 mg MDMA) or active dose group (75 mg, 100 mg, or 125 mg) doses during the open-label crossover. PTSD symptoms were reduced 1 to 2 months after MDMA-assisted psychotherapy, and symptom improvement continued at least 12 months post-treatment.
Mithoefer et al, 2019 [30]	Evaluating six phase 2 trials in a pooled analysis of 105 participants to determine the study design for phase 3 trials of MDMA-assisted psychotherapy for PTSD.	Patients with PTSD received control/placebo doses (0-40 mg) and active doses of MDMA (75-125 mg) during 2-3 8-hour psychotherapy sessions. All doses were well tolerated during experimental sessions and 7-day follow-up.
Feduccia et al, 2018 [7]	Exploring the role of memory reconsolidation and fear extinction through release of monoamines, hormones, and other downstream signaling molecules to dynamically modulate emotional memory circuits as underlying mechanisms of MDMA-assisted psychotherapy in PTSD patients.	By reducing activation in brain areas implicated in the expression of fear- and anxiety-related behaviors and increasing connectivity between the amygdala and hippocampus, MDMA may allow for reprocessing of traumatic memories and emotional engagement with therapeutic processes.
Barone et al, 2019 [31]	Qualitative study consisting of one-year follow-up of 19 out of 24 participants from a phase 2 clinical trial of MDMA-assisted psychotherapy for veterans, firefighters, and police officers suffering from chronic, treatment-resistant PTSD.	Interviews with participants showed that qualitatively significant and durable reductions in PTSD symptoms persisted more than 1 year after trial completion. Participants described improvement in self-awareness, relationships and social skills, substance use, and openness to continued therapy.
Feduccia et al, 2019 [32]	Review of Breakthrough Therapy Designation of MDMA-assisted psychotherapy data in comparison with traditional SSRI treatment data.	The active dose group (MDMA 75-125 mg, n=72) showed significant superiority to the control group (0-40 mg, n=31) via a drop in CAPS-4 total scores by -37.8 at 12 month follow-up. MDMA-assisted psychotherapy trials also had lower dropout rates as compared with sertraline and paroxetine trials.
Bershad et al, 2019 [33]	Measuring effects of MDMA in comparison with methamphetamine on pleasantness of affective touch and attentional bias towards positive facial expressions in 36 participants.	MDMA enhanced the pleasantness of social touch and showed clinical implications for treatment of psychiatric disorders. MDMA increased positive ratings of images with social content and emotional empathy. It also enhanced identification of positive expression while interfering with identification of negative expressions or both.

MDMA-Assisted Psychotherapy Showed Treatment Response for PTSD Symptoms and Significant Remission Status at Treatment Exit

**Figure 11:**
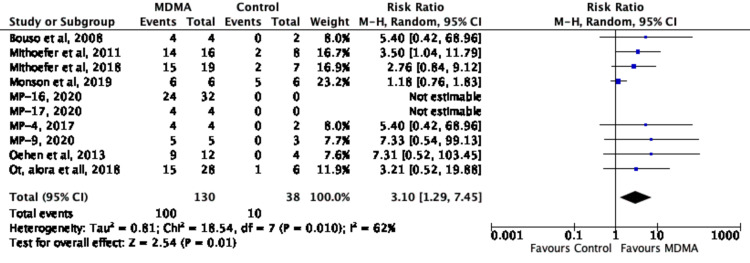
Forest Plot of Risk Ratio of Responders to MDMA (versus-control) Using Random Effects Meta Analysis MDMA: methylenedioxymethamphetamine [3,8,18-25]; MP-16: NCT03282123 [22]; MP-17: NCT03485287 [23]; MP-4: NCT01958593 [24]; MP-9: NCT01689740 [25]

After analyzing the data from ten studies [3,8,18-25], it was shown that participants in the experimental group who underwent MDMA assisted psychotherapy were more likely to show clinically significant responses compared to participants in the control group who underwent psychotherapy with or without a small dose of MDMA, as summarized in Figure 11. Table 2 shows that the number of MDMA sessions per study for the ten groups ranged between 1 and 3, with an average of 2.4 MDMA sessions among the experimental groups. The number of non-drug sessions completed during follow-up varied between the ten groups and consisted of psychotherapy during follow-up. Depending on the study, short-term follow-up occurred between 1 week and 5 months, while long-term follow-up occurred at 6-74 months, as seen in Table 2. 

Figure 11 shows the risk ratio of responders to MDMA in the ten studies [3,8,18-25], with 8 of them [3,8,18-21,24,25] having a calculable risk ratio at a 95% CI. Out of the eight risk ratios calculated, all 8 showed MDMA being favored. Each individual study is not statistically significant on its own. However, as seen in Figure 11, combining the samples of these ten studies provides a statistically significant analysis that favors MDMA use. Of the 130 participants in the experimental group, 100 participants were found to have responded positively, while 10 out of the 38 participants in the control group were found to have responded positively. 

Table 5 demonstrates that at the end of treatment, four studies found the rate of remission from PTSD to fall between 56% and 100%, with one of these studies [2] improving to a 67% remission rate up from 56% after long-term follow up at 12 months. The rate of remission for these four studies was based on the PTSD scoring scales of both SSSPTSD and CAPS-5, as summarized in Table 2. In order to be classified as being in remission, these patients had to no longer meet the criteria for a PTSD diagnosis as noted in Table 2. As seen in Table 2, the mean baseline severity of PTSD per study was as follows: Bouso et al.: 41.5 [18]; Jerome et al.: 85.1 [29]; MP-16 (NCT03282123): 45.42 [22]; MP-17 (NCT03485287) : 45.25 [23]. Table 2 shows that the mean baseline severity of PTSD for the groups ranged between 41.42 and 92, yielding 3 out of 4 of the studies discussed in Table 5 at a lesser severity compared to some of the studies. This means that on average the participants in the Bouso et al. [18], MP-16 (NCT03282123) [22], and MP-17 (NCT03485287) [23] had less severe PTSD compared to some of the participants in other studies, and may have had less severe symptoms to work through in order to achieve remission. 

**Table 5:** Remission at Treatment Exit with MDMA-assisted psychotherapy * at long term follow-up, at around 12 months, the remission rate increased from 56% to 67% (n=72) MDMA: 3,4-methylenedioxymethamphetamine MP-16: NCT03282123 [22]; MP-17: NCT03485287 [23]

Citation	No longer meeting PTSD Criteria (N, %)
Buoso et al, 2008 [18]	4, 67%
Jerome et al, 2020 [29]	60, 56%*
MP-16 [22]	24, 75%
MP-17 [23]	4, 100%

**Figure 12:**
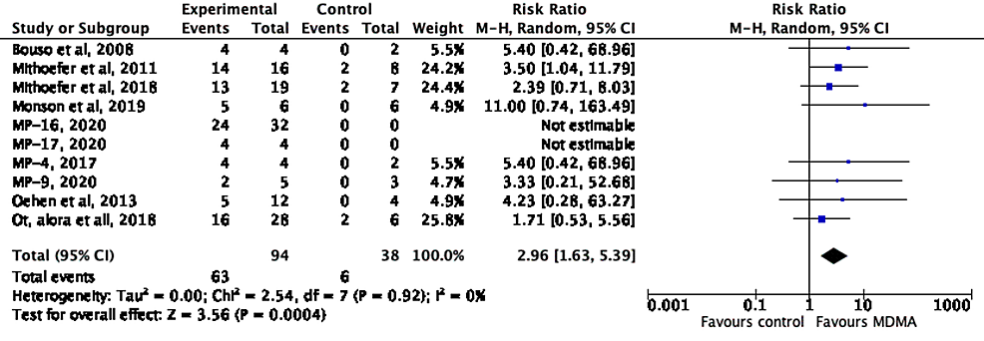
Forest plot for meta-analysis of remission of PTSD diagnostic status with MDMA-assisted psychotherapy MDMA: 3,4-methylenedioxymethamphetamine; PTSD: post-traumatic stress disorder [3,8,18-25]; MP-16: NCT03282123 [22]; MP-17: NCT03485287 [23]; MP-4: NCT01958593 [24]; MP-9: NCT01689740 [25]

Figure 12 shows the meta-analysis of those who went into remission from PTSD with MDMA-assisted psychotherapy in the 10 studies [3,8,18-25], with eight of them [3,8,18-21,24,25] having a calculable risk ratio at a 95% CI. Of the 130 patients from all ten studies, 100 individuals had gone into remission in the MDMA experimental group. Of the 10 studies with the control groups with all 38 individuals, 10 had gone into remission from PTSD. Because MP-16. (NCT03282123) [22] and MP-17 (NCT03485287) [23] did not have controls and thus were excluded from the analysis, their results of how many with PTSD went into remission after the intervention holds significant weight when comparing it besides the eight other studies. 

PTSD Symptom Scores Improved Following MDMA-Assisted Psychotherapy

**Figure 13:**
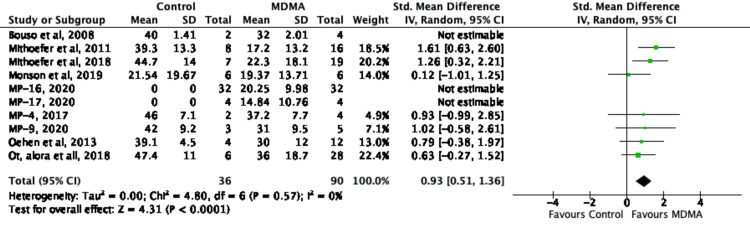
Forest plot of Standardized Mean Difference (SMD) of effect of MDMA (versus control) on PTSD symptoms score using random-effects meta analysis. MDMA: 3,4-methylenedioxymethamphetamine; PTSD: post-traumatic stress disorder [3,8,18-25]; MP-16: NCT03282123 [22]; MP-17: NCT03485287 [23]; MP-4: NCT01958593 [24]; MP-9: NCT01689740 [25]

Overall, Figure 13 shows that MDMA-assisted psychotherapy had a greater effect on improving CAPS-5 scores compared to the control groups. Bouso's 2008 [18] study was excluded from the analysis as part of a sensitivity analysis. Quantitatively, Figure 13 showed a low heterogeneity (χ^2^ = 4.80, I^2^ = 0%, p = .57), but the results from the random-effects model remained similar (standardized mean difference [SMD] .93 [95% CI: 0.51, 1.36]). The overall SMD for the change in PTSD symptom scores control versus MDMA intervention was .93 (95% CI: 0.51, 1.36),) indicating a large effect size (Figure 13). To further strengthen the result, the overall effect was calculated. The Z statistic (4.31) was used to calculate the p-value for hypothesis testing. The p-value calculated is (<0.0001) accepting the summary effect, making it statistically significant. Figure 12 also demonstrates the relationship between weight and precision as seen with study group Ot’alora et al. [3] with a weight of 22.4% in comparison to Bouso et al. [18] with a weight of 0.0%. The 95 % confidence interval, as well as the pooled result, does not overlap with the line of no effect passing through zero, which suggests that the data produced in the studies by Mithoefer et al. [19] and Mithoefer et al. [8] are statistically significant (p < 0.05). However, data produced in some studies [3,20-25] do have an overlap with the line of no effect, indicating that the data pooled is statistically insignificant with a p-value above 0.05. The funnel plot for this meta-analysis (Figure 8) was symmetric, indicating a low risk of publication bias.

Reduction in PTSD Symptom Scores Persist in Short and Long-Term Follow-Up After MDMA-Assisted Psychotherapy

**Figure 14:**
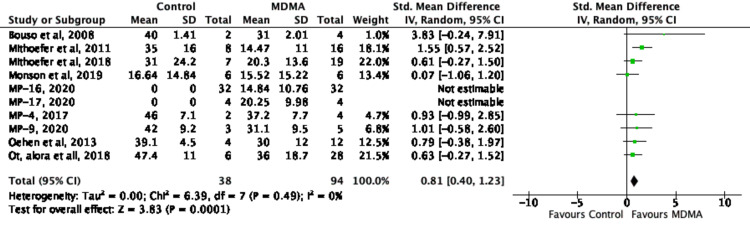
Forest plot of Standardized Mean Difference (SMD) of Pre-vs-Follow-up Effect of MDMA (versus control) on PTSD Symptoms Score using random-effects meta-analysis MDMA: 3,4-methylenedioxymethamphetamine; PTSD: post-traumatic stress disorder [3,8,18-25]; MP-16: NCT03282123 [22]; MP-17: NCT03485287 [23]; MP-4: NCT01958593 [24]; MP-9: NCT01689740 [25]

MDMA-assisted psychotherapy statistically maintained its therapeutic effect in patient follow-up, emphasizing the therapy’s potential longevity. This was measured by calculating the SMD comparing the effects of MDMA-assisted psychotherapy versus control on PTSD symptom score prior to, and after follow-up, as seen in Figure 14. The follow-up window, as can be seen in Table 2 lasted from 1-74 months. Homogeneity across studies was quantitatively demonstrated via a low level of heterogeneity (χ^2^ = 6.39, I^2^ = 0%, p = .49). The potential sustained effect of MDMA-assisted psychotherapy versus control was found to have a large effect size (SMD = 0.81; 95% CI: 0.40, 1.23), which supports a reduction in PTSD symptom scores and the other two measures of effect (RR of response, and SMD of pre versus post) (Figure 13). Effects from two of three sessions of MDMA-assisted psychotherapy were found to last several months, further strengthening the potential durability of this treatment. Table 4 shows that while all studies had a significant original pre-to-post treatment effect size, two studies, Bouso et al. [18] and Monson et al. [21], showed an increase in the pretreatment to long-term follow-up effect size. One study, Jerome et al. [29], however, showed a decrease in their long-term follow-up effect size. This was due to the number of patients who no longer met the PTSD criteria according to CAPS-4 total scores had increased to 67.0% at the time of follow-up compared to 56.0% at the time of treatment exit. Furthermore, as summarized in Figure 9, the funnel plot for this meta-analysis is also seen to be symmetrical, depicting a low risk of publication bias. 

Dose-Response Effects

Table 2 shows that the average number of MDMA sessions each study used is 2.4 with the majority of the studies conducting three MDMA sessions. Studies with three sessions tended to use higher doses of MDMA compared to the others. Doses ranged from 40-125mg of MDMA. The total number of sessions that included both MDMA-assisted psychotherapy and non-MDMA-assisted therapies is 15. In these studies, MDMA was administered in the presence of an observer, and subjects were often not redirected by observers which allows for participants' spontaneous experience and encourages them to process their emotions in a safe space. After analysis, Table 2 shows that all 10 studies had a baseline severity CAPS score reduction of at least 30%, with a noticeable positive effect on subjects administered higher doses of MDMA (75-125 mg). This positive effect ultimately placed subjects in the remission criteria after 12 months of follow-up. This supports the use of MDMA-assisted psychotherapy as beneficial for participants who experience chronic PTSD. Although there may be some bias present in the 10 studies because MDMA was used as an additive in addition to psychotherapy, most studies chose conventional treatment-resistant PTSD subjects to advance to MDMA-assisted psychotherapy. Those that did not choose participants who have been in therapy for PTSD and have not seen improvement may exhibit bias. 

Adverse Events

Of the 10 original studies analyzed and measured, only two studies, Monson et al. [21] and Mithoefer et al. [8], reported adverse events as shown in Table 2. The study by Monson et al. [21] reported no serious adverse events but cited the following side effects in six of the 12 participants: diminished appetite, anxiety, headache, jaw tightness, tinnitus, nausea, asthenia, fatigue, acute sinusitis, nasopharyngitis, upper respiratory tract infection, disturbance in attention, tremor, tics, dysuria, and erythema, which all resolved by the six-month follow-up period. The study by Mithoefer et al. [8] reported one possible drug-related serious event of depression with suicidal ideation. 

Symptomatology with Objective Outcomes 

While the quantitative findings from multiple MDMA-assisted psychotherapy trials have shown reliable symptom reduction via CAPS scores, PTSD symptom scores alone do not fully capture the potential benefits that can be gained from this treatment [32]. The CAPS-4 can assess the frequency and intensity of symptoms, but they are based on categorical questions. They cannot elucidate factors such as quality of life, impacted relationships, or increased substance abuse - which can all be a result of PTSD. It is important to try and gain a more subjective view of the treatment experience from the patients’ perspective in order to thoroughly understand the full benefit of the treatment. Several examples of this were described in Mithoefer et al. [26] and Barone et al. [31], in which a number of participants had minimal decreases in their CAPS scores, yet reported subjective improvement in mood and ability to function. Jerome et al. [29] and Monson et al. [21] was also used in this section to explore subjective responses to MDMA-assisted psychotherapy. These subjective analyses are not a substitute for the validated primary outcome measures, such as CAPS, but can provide important context in which the outcome data can be evaluated and understood (Table 6). 

**Table 6:** Symptomatology from Objective Outcomes MDMA: 3,4-methylenedioxymethamphetamine; CAPS: Clinician-Administered PTSD Scale

Citation	Clinical Trial	Related Articles	Findings
Mithoefer et al, 2011 [19]	NCT00090064	Mithoefer et al, 2013 [26]	Long term follow-up stabilization evident by CAPS scores and Self-Reported symptom scores. Also discussed improved cognitive function.
		Corey et al, 2016 [34]	Ensuic Utterances, in which speakers describe a change in their sense of themselves, seen as a predictor for CAPS score reductions and a hallmark of successful talk therapy during MDMA-assisted psychotherapy, during MDMA dose sessions.
		Jerome et al, 2020 [29]	Improvement in self-awareness, relationships in social skills, problem substance use, openness to continued therapy
Mithoefer et al, 2018 [8]	NCT01211405	Barone et al, 2019 [31]	Improvement in self-awareness, relationships in social skills, problem substance use, openness to continued therapy
Monson et al, 2019 [21]	NCT02876172		Increased relationship happiness, higher couples satisfaction index, improved sleep.

Mithoefer et al. [19] reported follow-up data to evaluate the long-term outcomes of their first completed trial of MDMA-assisted psychotherapy for chronic, treatment-resistant PTSD [19]. Part of this long-term follow-up (LTFU) was a questionnaire designed to specifically capture the perceived benefit or harm of the therapy and changes not addressed by the conventional standard outcome measures such as CAPS. Benefit and harm were measured with an ordinal scale of 1 (slight) to 5 (large), and the degree of persistence of perceived benefits was measured by 1 (small) to 5 (all). The questionnaire also included items that addressed the participants’ beliefs concerning the potential benefit of receiving an additional MDMA-assisted psychotherapy session, as well as any perceived changes in cognition after study participation. All 19 subjects reported a benefit from participating in the study (mean 4.3, median = 5, range = 3). Seventeen reported an increase in general well-being; 17 endorsed increased self-awareness and understanding; 15 reported less excessive vigilance; 15 endorsed less avoidance of people or places; 13 experienced fewer nightmares, flashbacks, or intrusive memories; 13 endorsed an increased ability to feel emotions; 12 reported reduced anxiety; 12 reported improved sleep; 11 endorsed improved relationships in general, while 10 endorsed improved relationships with spouse, partner, or other family members; 10 reported an enhanced spiritual life; 10 felt as if they were more involved in the community/world around them; nine reported improved mood; eight felt increased empathy for others, and six endorsed improved work performance. All answered “no” when asked if they felt they were harmed by participation in the study. All participants reported at least some benefit persisting (mean 3.8, median = 5, range = 3), with eight scoring 5, four scoring 4, three scoring 3, and four scoring 2. When asked about illicit drug use during the LTFU period, eight reported using cannabis ranging from “once” to “occasional,” and one reported using psilocybin-containing mushrooms but did not report frequency. All nine of these participants had begun using these substances prior to the initial study, so these were not considered new-onset uses. Also, only one participant reported using “ecstasy” in a quasi-therapeutic setting once. All participants answered “yes” when asked if they believed more MDMA sessions would be helpful for further treatment of their PTSD. Finally, when asked about cognitive function, memory, and concentration, 13 participants reported improvement while six reported no change. None indicated that these cognitive parameters had worsened or decreased.

Barone et al. [31] performed a long-term follow-up study of Mithoefer et al. [8] in which they examined the perceived benefits of MDMA-assisted psychotherapy from the participants' point of view, beyond quantitative symptom reduction. Semi-structured qualitative interviews were conducted during the participants’ long-term follow-up session one year after completing a phase 2 clinical trial that involved MDMA-assisted psychotherapy for 19 veterans, firefighters, and police officers with treatment-resistant PTSD and CAPS scores greater than 50. Their goal was to complement, clarify, and expand on the quantitative findings obtained from the CAPS-4 and LTFU questionnaire to identify key themes in participant experiences from before, during, and after the trial. They used the Interpretative Phenomenological Analysis (IPA) methodological approach, as it was considered to be optimal for conceptualizing the participants' personal experiences and perceptions of the treatment process. As stated by the authors, IPA is concerned with inquiry rather than hypothesis, and emphasizes the analysis of experience in its own terms rather than narrowing it into pre-established categories. From a quantitative point of view, 15 participants showed significant decreases in PTSD symptoms at the one-year follow-up, with a greater than 30% reduction in CAPS-4 scores from baseline. However, from a qualitative point of view, additional outcomes were explored that would be difficult to discern from PTSD scores alone, including self-awareness, relationships, and social skills, engagement in new activities, medication, and illicit substance use, and openness to future therapies. All 19 participants reported improved self-awareness and reported gaining valuable insights into their personal experiences as well as gaining deeper understanding during their LTFU interviews. Prior to the study, many of them had expressed how their experiences with trauma and PTSD had limited their ability to understand themselves or their issues. One year after treatment, subjective responses included feeling that the treatment had altered their perception of self and brought about more self-compassion, or that they were able to reality-test more effectively to avoid irrational negative thoughts, or that improved self-awareness gained in treatment helped them better understand and manage their PTSD symptoms. Twelve participants reported improved relationships, social skills, and social functioning as a benefit from participating in MDMA-assisted psychotherapy, and some even felt an increased sense of empathy for all people. Regarding engagement in new activities, many of the participants had expressed how their PTSD symptoms had led to isolation and limited participation in activities prior to the study, but at LTFU, all 19 reported that they were able to take more healthy and rewarding actions in their lives, regardless of the degree to which their symptoms had changed. Many credited the study as the main reason for a newfound feeling of motivation to try new things, as well as a renewed enthusiastic drive to engage with life. As for medications and substance use, all 19 participants had been prescribed medications for PTSD prior to the study, and nine recalled extensive substance use to help cope with their symptoms. At LTFU, most had reduced their reliance on medications over the course of the treatment, and 13 reported that reduction in PTSD symptoms and meaningful experiences during the study contributed to this. Some who had used substances reported a marked decrease in urge to use, or reflected how the treatment helped them appreciate the enduring changes brought about by the treatment, rather than the short-term relief offered by substance use. Fourteen of the 19 participants described that their experience with MDMA-assisted psychotherapy had helped them feel more open to exploring other treatment modalities, despite all having experienced little to no improvement in prior pharmacological or psychotherapeutic treatments before the study. Experiences included a breaking down of personal defenses, the beginning of a healing process, and more capability to work through difficult memories. 

Jerome et al. [29] conducted a study in which they extended the follow-up of participants across six phase 2 clinical trials involving MDMA-assisted psychotherapy [3,8,10,19,20,33]. Their primary aim was to expand upon the initial findings by pooling data from all six trials to examine the long-term effects of MDMA-assisted psychotherapy on PTSD symptoms and other benefits/harms at least one year post-treatment. This assessment included a long-term follow-up questionnaire (LTFUQ) to assess subjective participant response to benefits and harms from participation in the phase 2 trials as a secondary measure. The LTFUQ occurred approximately 12 months after the last MDMA session in five of the trials, and in one (MP-1) the LTFUQ occurred over an average of 3.8 years as it was added later as a study amendment. The questionnaire would track elements of recovery that were not included in measures of PTSD symptoms, such as changes in interpersonal relationships, personal growth, and spirituality. The strength of benefits and harms were scored by the participants on a five-point Likert scale, with 1 = slight and 5 = large or severe. A question on the duration of benefits/harms was also added, as well as an inquiry on substance use and suicidal ideation. 97.6% of participants across studies reported experiencing benefits, and 92.2% of those reported that those benefits had lasted. 53.2% indicated large benefits that lasted or continued to grow. Seven participants reported harm (8.4%), with only two reporting the harm still present at 12-month follow-up. Six rated the harm as 3 or lower and one reported a score of 4, with none reporting severe harm. All seven of these participants reported at least one benefit. 84.3% of respondents reported increased general well being; 71.1% endorsed fewer nightmares, flashbacks, or intrusive memories; 73.5% reported increased ability to feel; 68.7% reported fewer avoidance symptoms; 68.7% endorsed reduced anxiety; 72.3% endorsed less hypervigilance; 66.3% reported improved sleep; 61.5% reported improved relationships with spouse, partner, or other family members while 66.3% reported improved relationships in general; 61.5% endorsed improved spiritual life; 89.2% reported increased self-awareness and understanding; 61.5% reported increased empathy for others; 61.5% endorsed improved mood; and 53% reported being more involved in the community or world around them. 94% of participants felt that additional MDMA sessions would be helpful. Furthermore, proportions of participants who reported positive suicidal ideation decreased from 60% at baseline to 24% at LTFU. Regarding substance use, self-reported alcohol consumption since study enrollment decreased among 40% of participants, stayed the same for 30.9%, and increased for two (3.6%). Some participants reported greater marijuana use at LTFU (18.2%) while others reported less (18.2%); 6% reported their marijuana use as staying the same. At baseline, 32 of 107 participants (29.9%) had reported at least one prior use of ecstasy. At the LTFU, eight (9.6%) reported using ecstasy or MDMA between treatment exit and LTFU for therapeutic or recreational purposes. Six of the eight had used it prior to the study, while two had not.

Corey et al. [34] conducted a study based on audio recording data of twenty subjects from Mithoefer et al. [19] with CAPS scores greater than 50. This analysis took a novel approach by simultaneously analyzing psychotherapy, psychopharmacology, and discourse-level linguistics by examining the initiation of discourse topics by participants using MDMA or placebo. The recordings used were only for the first psychotherapeutic session, as this would show the “cleanest” effect of MDMA with the least accumulated effects of psychotherapy. “Ensuic'' utterances described a change in the participants’ sense of themselves and could be considered a sign of successful talk therapy during MDMA-assisted sessions, as well as a predictor for CAPS score reductions. These occurred in narrative formats and were announced as subjective experiences. Topic initiations of other people’s feelings, usually the therapist’s, were described as “empathic,” and initiations of a desire for or pleasure in touch were described as “entactic.” Subjects who received MDMA (125 mg) produced many more scored ensuic, empathic, and entactic utterances compared to those who received placebo. A significant relationship was found between higher number of scored utterances and lower CAPS scores measuring PTSD severity after the treatment by Pearson’s r (r = -0.506, p = 0.023, n = 20). The presence of MDMA significantly increased the quantity of ensuic, empathic, and entactic topic initiation utterances in a group of subjects with treatment-resistant PTSD, and such utterances appear to reflect cognitive processes important to improvement in the participants’ mental health as shown by their posttreatment CAPS scores. 

Monson et al. [21] conducted an uncontrolled trial involving six couples with varying levels of baseline relationship satisfaction in which one partner was diagnosed with PTSD. It involved 15 sessions of cognitive-behavioral conjoint therapy (CBCT) for PTSD over seven weeks with MDMA sessions timed to synergize with the CBCT interventions. There were two sessions in which both members of the couple were administered MDMA. Each partner was given 75 mg of MDMA during the first MDMA session, and 100 mg in the second session, with an optional supplemental half-dose 1.5 hours later for both sessions as this could prolong the therapeutic window of MDMA effects. Each member of the couple was scheduled for assessment at pre-treatment, mid-treatment, post-treatment, and three and six-month follow-ups. Participants also completed assessments of self-reported and partner-reported PTSD symptoms and overall relationship happiness at the assessment points, as well as at each treatment session. They were assessed for eligibility using the Structured Clinical Interview for DSM-5 (SCID-5), and CAPS-5 was used as the primary outcome measure for PTSD symptoms. The PTSD Checklist for DSM-5 (PCL-5), patient and partner versions, and Couples Satisfaction Index (CSI) were used for self-reported primary outcomes. There were significant improvements in clinician-assessed, patient-rated, and partner-rated PTSD symptoms. Growth curve modeling revealed significant and sustained improvements in CAPS scores at post-treatment and follow-ups. From the participants’ subjective experiences, significant improvements in overall patient and partner relationship satisfaction were found. Of the three partners who were relationally distressed at baseline, only one was distressed at post-treatment and follow-ups, and this was the partner of the only participant who retained their PTSD diagnosis. This was also the only patient who had been relationally satisfied at baseline but dissatisfied at post-treatment and follow-up. Furthermore, growth curve models revealed overall improvements in patient depression, sleep, emotional regulation, and overall trauma-related beliefs.

Discussion

The aim of this article was to compute a meta-analysis and conduct a systematic review of the effects of MDMA on post-traumatic stress disorder, as well as to discuss the potential benefits and adverse events relative to dosing and stability of treatment. The results from our meta-analysis support the use of MDMA as an adjunctive treatment to psychotherapy for chronic, treatment-resistant PTSD. The overall risk ratio (RR) for a clinical response was significantly higher in the experimental group (RR = 3.10, 95% CI: 1.29, 7.45), showing that after treatment the participants had a significantly increased likelihood of clinical response for PTSD symptoms. Regarding remission, a random-effects meta-analysis showed an overall RR for remission to also be higher in the experimental group (RR = 2.96, 95% CI: 1.63, 5.39). MDMA-assisted psychotherapy also resulted in a significant reduction in PTSD symptoms, with an SMD of 0.93 for change in symptom scores from before to after treatment (SMD = 0.93, 95% CI: 0.51, 1.36). Furthermore, this reduction in symptoms was maintained in the extended follow-up periods which ranged from 2-32 months (SMD = 0.81, 95% CI: 0.40, 1.23). We also examined the use of long-term follow-up questionnaires during the follow-up periods to gain insight into the subjective experiences of the participants to thoroughly understand the full benefit of the treatment. Interestingly, there were several examples across the studies in which participants did not have a significant reduction in their PTSD scores yet felt that the treatment was helping improve how they felt and were functioning. 

Research into MDMA-assisted psychotherapy, as well as other nontraditional treatment options for PTSD (e.g., vagal nerve stimulation, stellate ganglion blockade), has occurred because of the non-response rates that were observed with traditional therapies [35]. While CBT with prolonged exposure therapy is effective in some patients, it takes time and considerable expertise [36], and the efficacy of SSRIs in the treatment of PTSD has recently come into question [37,38]. Furthermore, less than half of patients with PTSD either seek or receive treatment, and for those that do, the dropout rate is approximately 24% [36]. Feduccia et al. [32] compared data that was used for the approval of the only two FDA-approved oral medications for the treatment of PTSD, sertraline and paroxetine, with the data for MDMA-assisted psychotherapy that was submitted for its successful breakthrough therapy application. Primary efficacy evaluation in CAPS scores (CAPS-2 for the SSRIs, CAPS-4 for MDMA-assisted psychotherapy) showed a significant effect favoring MDMA over the comparison group (p < 0.001) with a large between-group effect size (Cohen’s d effect size = 0.9) that was approximately double that of paroxetine (0.45-0.56) and triple that of sertraline (0.31-0.37). It also showed a greater mean decrease in CAPS total scores 1-2 months after blinded experimental sessions, with -26.2 units versus paroxetine (-6 to -14 units) and sertraline (-6.8 to -9.8 units). Furthermore, the authors examined outcome measures for participants in the six MAPS-sponsored phase 2 MDMA-assisted psychotherapy trials that previously had non-responsive SSRI treatment. Out of 105 participants, 17 had taken paroxetine, 35 had taken sertraline, and 12 had tried both. Thirty-eight of these participants were randomized to the active group, and 20 (52.6%) of them no longer met the criteria for PTSD at the primary endpoint. The mean drop in CAPS-4 scores was -40.1 for prior paroxetine participants and -35.04 for participants who had previously taken sertraline [33]. Regarding first-line psychotherapies for PTSD, 40-60% of patients do not respond adequately [7]. Lee et al [37] reported effect sizes from meta-analyses to show that trauma-focused psychotherapy had a greater effect size (-0.96) than medication (-0.44). In comparing MDMA-assisted psychotherapy to trauma-focused psychotherapy, the magnitude of the effect of MDMA-assisted psychotherapy is within the range of the first-line trauma-focused therapies (0.9) [33]. While meta-analyses and randomized controlled trials generally seem to show the superiority of trauma-focused psychotherapy over pharmacotherapy and other psychotherapies, randomized trials comparing them to MDMA-assisted psychotherapy should still be done to show which is superior in efficacy and tolerance. However, based on the results of our meta-analysis, as well as the high dropout rate present with conventional treatments, the results are promising.

Several possible mechanisms of action have been postulated as to how the addition of MDMA during psychotherapy causes a reduction in PTSD symptoms in patients. From a neurobiological perspective, findings from both clinical and nonclinical studies suggest a unique mechanism in which MDMA affects the brain regions involved in emotional memory processing, fear extinction learning, memory reconsolidation, and cognition [29,33]. MDMA promotes the release of serotonin (5-HT), dopamine (DA), and norepinephrine (NE) in the brain by reversing membrane-bound transporters and blocking reuptake in the mesolimbic pathway [7]. For proper emotional and cognitive behavior, there must be a proper balance of these three neurotransmitters in the mesolimbic, as well as the mesocortical pathway. For example, low 5-HT is implicated in major depression, and there is an overlap in depressive symptoms with PTSD, including negative mood, difficulty with sleep, loss of interest, guilt, concentration difficulties, and suicidal ideations. The SSRIs paroxetine and sertraline are first-line pharmacotherapy for major depression and PTSD, yet we have discussed the overall lack of efficacy of these medications in PTSD. The effects of dopamine in these pathways are also significant. In the mesolimbic pathway, DA is an important neurotransmitter in circuits from the ventral tegmentum of the midbrain to the amygdala and hippocampus (limbic system), as well as to the nucleus accumbens (reward center). The limbic system is a crucial region involved in the pathology of PTSD, as the hippocampus is involved in the long-term potentiation of memory and learning, and plays a part in the intrusive reminders of the trauma that PTSD patients experience, such as flashbacks, memories, and nightmares. The amygdala is directly involved in emotional regulation, including fear. The role of the amygdala in fear and anxiety is well documented, and PTSD seems to be associated with chronic disinhibition of the amygdala, which may explain symptoms such as exaggerated startle reflex and mobilization of the autonomic nervous system in these patients and may be directly implicated in their emotional and autonomic shifts [39]. In the mesocortical pathway, low DA in circuits that project from the ventral tegmentum to the prefrontal cortex are associated with decreased attention and cognition, as well as negative symptoms, all of which occur in PTSD patients. Norepinephrine** **from the locus coeruleus is involved with physiological responses to stress and panic. Exacerbated sympathetic autonomic responses such as increased blood pressure and heart rate to acute stress are prevalent in PTSD, as are arousal symptoms such as increased startle response, hypervigilance, sleep disturbances, and anger outbursts. Studies show that military veterans with chronic PTSD have higher cerebrospinal fluid concentrations of NE at baseline in unstressed conditions compared to healthy controls [39]. However, even as MDMA may be affecting these brain regions and neurotransmitters acutely, there are additional long-term positive effects, as many of the studies in our meta-analysis show during the follow-up (LTFU) periods. MDMA-assisted psychotherapy is effective in reducing PTSD symptoms after only two to three administrations of MDMA in conjunction with several non-drug therapy sessions producing long-lasting PTSD remission [20,26]. It is in seeking to understand these long-lasting effects of MDMA-assisted psychotherapy that the unique mechanism mentioned earlier has been postulated.

Fear extinction is described in classical conditioning models and is at the heart of exposure therapies that used to treat PTSD. An unconditioned stimulus, in the case of PTSD being a traumatic experience, causes a conditioned fear response when an individual is exposed to reminders or cues of the traumatic event, resulting in a conditioned fear reaction. This physiological fear response includes symptoms of stress and anxiety, avoidance behaviors, and exaggerated startle [7]. Extinction refers to repeated real or imagined exposure to the conditioned stimulus without encountering the original unconditioned stimulus to reduce or eliminate the conditioned fear response while leaving the original trauma memory intact. Exposure therapies used in PTSD aim to extinguish fear by presenting the conditioned stimulus in imagined narratives or reality-based situations while the patient feels they are in a safe setting. Encountering the past while on MDMA in a safe and supportive setting may extinguish the fear associated with traumatic events through the same processes of exposure-based therapies, but in a more rapid and effective way due to the physiological effects of MDMA [7]. Young et al. [40] demonstrated that MDMA facilitates fear extinction and neurobiological processes associated with extinction learning in rats. MDMA treatment before extinction training-induced long-term reductions in conditioned fear that persisted even when the fear-eliciting stimulus was presented in a novel context. Furthermore, they and another study done by Esber et al. [41] were able to enhance extinction by directly infusing MDMA into the basolateral complex of the amygdala (BLA) or infralimbic subregion of the medial prefrontal cortex (mPFC). The ventromedial prefrontal cortex (vmPFC) projects directly to the amygdala and is implicated in the processing of fear, as it is thought to provide inhibitory input to the amygdala, making it critical in the regulation of amygdala activity. Studies have demonstrated that when PTSD patients are exposed to reminders of traumatic events, they show diminished hemodynamic responses in the vmPFC, yet exaggerated responses in the amygdala, suggesting that PTSD is associated with overactivation of the amygdala due to a lack of vmPFC inhibitory control [41]. Extinction enhancement by MDMA coincided with increased markers of neuronal activity in the amygdala and mPFC, as well as increased expression of BDNF in the amygdala. According to the authors, recovery from PTSD and extinction learning both depend on BDNF, as individuals with gene variants that result in reduced release of BDNF are predisposed to developing PTSD and are less responsive to exposure-based therapy [40]. Disrupting BDNF signaling in the BLA completely abolished MDMA’s effect on extinction, suggesting that MDMA enhances fear extinction learning in a BDNF-dependent manner [40,41]. In response to Young et al. [40], MAPS has sponsored a similar study in humans to assess whether giving MDMA prior to the extinction of conditioned fear can reduce startle responses during extinction recall [7]. 

Memory reconsolidation describes a neuroplastic process where reactivation of a stored memory in the brain can make the memory transiently labile. During the time it takes for the memory to restabilize (reconsolidate) the memory can either be reduced by an amnesic agent or enhanced by memory enhancers [7]. The change in memory expression is related to changes in the brain function in processing long-term memory. Hypothetically, MDMA induces this retrieval-induced plasticity during psychotherapy, enabling traumatic memories to be updated with new information in a safe, therapeutic setting. This creates a mismatch, or “prediction error,” of the traumatic event [7], in which the patient can retrieve the memory with a changed, more positive, emotional context. The hippocampus is responsible for forming new memories, but the amygdala is responsible for the emotional context of the memory, telling us whether the memory was good or bad. The mesocortical DA projection to the amygdala is specifically associated with initial destabilization of the memory, allowing it to become temporary labile and amenable to neuroplastic modification and stabilization [42], or emotional memory reprocessing. After destabilization, this reprocessing is thought to occur due to the positive affective state and prosocial effects brought forth by MDMA, perhaps through increased 5-HT release, in the safe and supportive setting of psychotherapy [7]. Interestingly, research including brain imaging studies by Carhart-Harris et al. [43], showed that MDMA increased resting-state functional connectivity between the hippocampus and amygdala in healthy subjects, but no such studies have been performed thus far in PTSD patients under the influence of MDMA. Further research with brain imaging in PTSD patients while taking MDMA, especially fMRI, would be helpful in clarifying if the changes in healthy subjects observed by Carhart-Harris et al. definitively apply to the PTSD population as well. 

While research into the neurobiological aspects of MDMA-assisted psychotherapy should and will continue, the studies included in our meta-analysis and elsewhere thus far support the growing body of evidence showing the efficacy of the treatment in PTSD patients. Perhaps the best proof of this so far is that the therapy was granted Breakthrough Therapy Designation (BTD) by the FDA and it has entered phase 3 clinical trials. Furthermore, subjective explanations by researchers and especially the participants support the seemingly positive effect of this treatment. Clinical observations and subjective reports of MDMA’s prosocial effects strongly suggest that the enhanced therapeutic relationship that occurs during MDMA-assisted psychotherapy is an important mediator of its positive outcomes [7]. The preparation sessions and integrative sessions appear to be important for safety and therapeutic effect, and the close follow-up that occurs seems to support further processing of emotions and integration of any cognitive shifts that may occur [19]. MDMA is administered in a setting that is designed to enhance and support the therapeutic effects of the compound, and when administered in a therapeutic setting, MDMA appears to increase the tolerability and effectiveness of psychotherapy [29]. Participants often report experiencing lasting personal benefits and enhanced quality of life that extend beyond reduced, quantifiable symptom scores [31]. If MDMA-assisted psychotherapy successfully clears phase 3 trials, it is our hope that this promising modality can help improve the symptoms and morbidity experienced by the significant PTSD population that has not responded to conventional treatments thus far. 

Finally, we explored the adverse events relative to dosing and stability of MDMA-assisted psychotherapy. It would only be natural for those outside of the scientific community or unfamiliar with research into the subject to question the safety of the use of what is seen by many as an illicit drug in the treatment of PTSD. Such treatments often have to face political hurdles as well. MDMA is a phenylethylamine with structural similarities to both amphetamine and mescaline [44]. Concern over its abuse and subsequent possible neurotoxicity led to its assignment as a Schedule I agent by the U.S. Drug Enforcement Agency in 1985 [44]. Since then, there has been an ongoing debate about its therapeutic effects and whether the neurotoxic effects seen after high or repeated doses are a cause for concern [44]. While the use of illicit MDMA, or “ecstasy,” may result in overdose with the side effects mentioned earlier [4], trials and studies involving MDMA-assisted psychotherapy have been shown to be relatively safe. The side effects reported by Monson et al. [21] of diminished appetite, anxiety, headache, and jaw tightness that self-resolved parallel the ones reported by Feduccia et al. [32] wherein patients were administered an active dose of 75-125 mg. The study by Monson et al. [21] used 75 mg or 100 mg with an additional half dose in some patients resulting in a cumulative dose reaching 107.5 mg to 150 mg. The study by Mithoefer et al. [8] that reported one case of depression with suicidal ideation reported dosing up to 125 mg, however, they were unable to ascertain whether this was drug related. This finding as a side effect has scarcely been reported in the literature, however, symptoms like depressed mood and psychosis have been reported [44]. The side effects including difficulty concentrating, jaw clenching, tremor, weakness, fatigue, and lack of energy have also been previously reported in another study in patients given a dose of 1.7 mg/kg [44]. This study found that dosages ranging from 40 mg to 187.5 mg were effective in resolving the symptoms of PTSD as evidenced by a decrease in the CAPS score to varying degrees (Table 2). These findings suggest that the range of 40 mg to 187.5 mg can be safely used in patients while expecting the side effects of anxiety, diminished appetite, headache, and jaw tightness that will self-resolve. 

Strengths and limitations

While our findings are promising, there are several limitations that ought to be highlighted. The most notable is the small sample sizes, the heterogeneity of experimental groups, MDMA dosing, duration of treatment, control groups, and follow-ups. The quality of the evidence from each study ranged from moderate to high using the Cochrane Risk of Bias Tool. Although, it is a given that in most cases the clinicians and participants were aware of the treatment, despite being double-blinded, due to the overt effects of MDMA. We were able to identify two studies with high-risk domains; Bouso et al. [18] had incomplete outcome data and MP-17 (NCT03485287) (2020) [23] had a lack of blinding of outcome assessments, as seen in Figure 6. 

Additionally, given that all 10 studies [3,8,18-25] required that participants had to have prior experience with MDMA, it would have been difficult for these participants to be truly assigned a placebo group due to the dramatic undeniable effects of MDMA. Subjects who felt like they were not being administered MDMA were more likely to drop out of the project due to lack of effect. In discussing heterogeneity of assessment tools, there was the usage of both CAPS-4 and CAPS-5 which could cause data bias due to inaccurate interpretation. For instance, CAPS-4 symptom severity ratings are based on two separate scores of frequency and intensity of symptoms whereas in the new CAPS-5 symptom severity ratings have been combined to a single severity score. 

The findings of this study may not be generalizable to the PTSD population at large given that this study specifically targeted patients with treatment-resistant PTSD and a prior history of MDMA use, though this does warrant further investigation. Additionally, the average age of participants based on six studies [3,10,18-21] was 40.6 years old, with four of the studies [22-25] listing the age only as 18-21 years or above. This could mean that the average participant more closely represented a certain cohort, and the effects may not as readily help those that are outside of those ranges. Five of the other studies [3,10,19-21] listed that some of the participants had comorbidities such as depression and anxiety. On average, 78.2% of participants in the five listed studies [3,10,19-21] had comorbid depression, while 38.2% had comorbid anxiety. This high level of comorbid depression may have been a confounder in participants' treatment and recovery of their PTSD, as it is unknown whether or not they received any treatment for these underlying problems as well.

Another notable limitation is underlined in the study by Monson et al. [21] where they reference their lack of a "control" group. Although it was deemed a lack of a control for their study, the experimental group mirrored only one eligible couple, where one partner met the diagnostic criteria for PTSD and the other did not. In this perspective, the total participants (six couples, 12 participants) consisted of six diagnosed with PTSD and six not. They were all administered MDMA, but the measured scales and checklists helped quantify the data in measuring outcomes for PTSD reductions and improvements in the couples scales. The limitation to this study is also due to the fact that it was an uncontrolled trial, but it was designed in such a way to show improvements in the patients who had PTSD reduction scores post-treatment as its strength. Furthermore, the heterogeneity across the control groups in the studies included proved challenging to ascertain the true degree of improvement of symptoms in comparison to the experimental group.

## Conclusions

We systematically reviewed and meta-analyzed randomized and quasi-randomized and uncontrolled trials, measuring the effectiveness and safety of MDMA-assisted psychotherapy for treating chronic, treatment-refractory PTSD. We identified 10 moderate-quality trials demonstrating that MDMA-assisted psychotherapy was associated with significant improvements in PTSD symptoms following intervention that extended long-term with few reported adverse effects. 

In conclusion, our work suggests that MDMA-assisted psychotherapy is a safe, effective and durable treatment for individuals with PTSD. Phase 3 clinical trials are near completion, which will serve to increase the validity of this evidence. With all the evidence collected and the positive outcomes demonstrated, it is also recommended to advance using MDMA-assisted psychotherapy as a method of treatment for other patients with psychiatric illnesses. Based on the chemical makeup of MDMA and the neurotransmitters they target, exploration of this treatment method for depression, bipolar disorder, anxiety and other anxiety related disorders may potentially also hold weight for positive outcomes relevant to potentially remission, effective treatment response in shorter duration and improve therapeutic alliances with patients.

## Appendices

Coding Guide

Study Variables Coded

The following variables were coded:

1. Study name

2. First author

3. Funding source (all studies were funded by the Multidisciplinary Association of Psychedelic Studies [MAPS])

4. Country of study

5. Study type (e.g., interventional, observational, other)

6. Gross description of the study design

7. Description of recruitment measures

8. Description of the study blinding and randomization

9. Screening tests used

10. Type of treatment the intervention is being compared to (e.g., placebo, standard therapy, no treatment, other)

11. Description of primary and secondary outcome measures

12. MDMA regimen (dose, route, frequency, source)

13. If study was psychotherapy assisted or not (yes/no)

14. Beneficial outcomes relative to dosing

15. Description of statistical method(s) used

16. Description of safety/adverse events relative to dosing

17. Completion of scheduled treatment

18. Duration of intervention and follow-up period

19. Percentage of participants who no longer met PTSD diagnostic criteria following intervention

20. Percentage of participants who followed up after completion of trial

21. Scales used in follow-up period

22. Description of effect size

Demographic Variables Coded

The following demographic variables were coded:

1. Percentage of participants who were female (%) and/or male (%)

2. Age (mean, range)

3. Percentage of participants belonging to different ethnic groups (%)

4. Percentage of participants who were single, married, divorced/separated, or widowed (%)

5. Percentage of participants who were unemployed, on disability, employed part-time, employed full-time, or retired (%)

6. Qualitative description of participants

Psychiatric History Variables Coded

The following psychiatric history variables were coded:

1. Percentage of participants with a history of alcohol, cannabis, tobacco, MDMA, psilocybin, illicit drug, or “other” drug use (%)

2. Mean lifetime number of prior times MDMA was used (range)

3. Mean number of months of duration of PTSD diagnosis (n, SD)

4. Mean number of months of prior psychotherapy for PTSD (n, SD)

5. Percentage of participants with a history of a mood, anxiety, or eating disorder (%)

6. Percentage of participants with a particular index trauma (childhood sexual abuse, other sexual abuse, accident, medical treatment, illness, natural disaster, crime-related, military service-related and first responder related (%)

7. Percentage of participants who were taking prescribed medications at the time of study enrollment (%)

## References

[1] (2021). What is MDMA?. https://www.drugabuse.gov/publications/research-reports/mdma-ecstasy-abuse/what-mdma.

[2] (2021). Inverse: MDMA steps closer to FDA approval as a drug, but now it needs to leap. https://maps.org/articles/6094-inverse-mdma-steps-closer-to-fda-approval-as-a-drug.

[3] Ot'alora G M, Grigsby J, Poulter B (2018). 3,4-Methylenedioxymethamphetamine-assisted psychotherapy for treatment of chronic posttraumatic stress disorder: a randomized phase 2 controlled trial. J Psychopharmacol.

[4] (2021). What are the effects of MDMA | National Institutes of Health. National Institutes of Health.

[5] (2021). PTSD and DSM-5. https://www.ptsd.va.gov/professional/treat/essentials/dsm5_ptsd.asp.

[6] Bahji A, Forsyth A, Groll D, Hawken ER (2020). Efficacy of 3,4-methylenedioxymethamphetamine (MDMA)-assisted psychotherapy for posttraumatic stress disorder: A systematic review and meta-analysis. Prog Neuropsychopharmacol Biol Psychiatry.

[7] Feduccia AA, Mithoefer MC (2018). MDMA-assisted psychotherapy for PTSD: are memory reconsolidation and fear extinction underlying mechanisms?. Prog Neuropsychopharmacol Biol Psychiatry.

[8] Mithoefer MC, Mithoefer AT, Feduccia AA (2018). 3,4-methylenedioxymethamphetamine (MDMA)-assisted psychotherapy for post-traumatic stress disorder in military veterans, firefighters, and police officers: a randomised, double-blind, dose-response, phase 2 clinical trial. Lancet Psychiatry.

[9] Kuypers KP, Wingen M, Heinecke A, Formisano E, Ramaekers JG (2011). MDMA intoxication and verbal memory performance: a placebo-controlled pharmaco-MRI study. J Psychopharmacol.

[10] Baumann MH, Rothman RB (2009). Neural and cardiac toxicities associated with 3,4-methylenedioxymethamphetamine (MDMA). Int Rev Neurobiol.

[11] Kirkpatrick MG, Baggott MJ, Mendelson JE, Galloway GP, Liechti ME, Hysek CM, de Wit H (2014). MDMA effects consistent across laboratories. Psychopharmacology (Berl).

[12] Ruis C, Postma A, Bouvy W, van der Ham I (2015). Cognitive disorders after sporadic ecstasy use? A case report. Neurocase.

[13] Bedi G, Cecchi GA, Slezak DF, Carrillo F, Sigman M, de Wit H (2014). A window into the intoxicated mind? Speech as an index of psychoactive drug effects. Neuropsychopharmacology.

[14] Blake DD, Weathers FW, Nagy LM, Kaloupek DG, Gusman FD, Charney DS, Keane TM (1995). The development of a clinician-administered PTSD scale. J Trauma Stress.

[15] Review Manager . https://training.cochrane.org/online-learning/core-software-cochrane-reviews/revman (2021). RevMan | Cochrane Training. Cochrane RevMan 5.4.

[16] Sterne JAC, Savović J, Page MJ (2019). RoB 2: a revised tool for assessing risk of bias in randomised trials. BMJ.

[17] Sterne J A, Hernán M A, McAleenan A, Reeves BC, Higgins JPT Chapter 25: Assessing risk of bias in a non-randomized study. Cochrane Handbook for Systematic Reviews of Interventions.

[18] Bouso JC, Doblin R, Farré M, Alcázar MA, Gómez-Jarabo G (2008). MDMA-assisted psychotherapy using low doses in a small sample of women with chronic posttraumatic stress disorder. J Psychoactive Drugs.

[19] Mithoefer M, Wagner M, Mithoefer A (2011). The safety and efficacy of 3,4-methylenedioxymethamphetamine- assisted psychotherapy in subjects with chronic, treatment-resistant posttraumatic stress disorder: the first randomized controlled pilot study. J Psychopharmacol.

[20] Oehen P, Traber R, Widmer V, Schnyder U (2013). A randomized, controlled pilot study of MDMA (± 3,4-Methylenedioxymethamphetamine)-assisted psychotherapy for treatment of resistant, chronic Post-Traumatic Stress Disorder (PTSD). J Psychopharmacol.

[21] Monson C, Holland J, Mithoefer M (2021). MDMA-assisted and cognitive-behavioral conjoint therapy (CBCT) in dyads with one member with chronic PTSD. Clinicaltrials.gov.

[22] MP-16. (2017, October October (2021). Open label multi-site study of safety and effects of MDMA-assisted psychotherapy for treatment of PTSD. https://clinicaltrials.gov/ct2/show/NCT03282123.

[23] MP-17. (2018, April April (2021). Study of safety and effects of MDMA-assisted psychotherapy for treatment of PTSD. https://clinicaltrials.gov/ct2/show/NCT03485287.

[24] (2021). Randomized, double-blind, controlled of MDMA-assisted psychotherapy in 12 subjects with PTSD. https://clinicaltrials.gov/ct2/show/NCT01958593.

[25] MP-9. (2013 (2021). Randomized, double-blind, active placebo-controlled pilot study of MDMA-assisted psychotherapy in people with chronic PTSD. https://clinicaltrials.gov/ct2/show/NCT01689740.

[26] Mithoefer MC, Wagner MT, Mithoefer AT (2013). Durability of improvement in post-traumatic stress disorder symptoms and absence of harmful effects or drug dependency after 3,4-methylenedioxymethamphetamine-assisted psychotherapy: a prospective long-term follow-up study. J Psychopharmacol.

[27] Mithoefer M (2016). A Manual for MDMA-Assisted Psychotherapy in the Treatment of Posttraumatic Stress Disorder. http://www.maps.org/research/mdma/mdma-research-timeline/4887-a-manual-for-mdma-assisted-psychotherapy-in-the-treatment-of-ptsd.

[28] (2021). MDMA-assisted therapy study protocols. https://maps.org/research/mdma.

[29] Jerome L, Feduccia AA, Wang JB (2020). Long-term follow-up outcomes of MDMA-assisted psychotherapy for treatment of PTSD: a longitudinal pooled analysis of six phase 2 trials. Psychopharmacology (Berl).

[30] Mithoefer MC, Feduccia AA, Jerome L (2019). MDMA-assisted psychotherapy for treatment of PTSD: study design and rationale for phase 3 trials based on pooled analysis of six phase 2 randomized controlled trials. Psychopharmacology (Berl).

[31] Barone W, Beck J, Mitsunaga-Whitten M, Perl P (2019). Perceived benefits of MDMA-assisted psychotherapy beyond symptom reduction: qualitative follow-up study of a clinical trial for individuals with treatment-resistant PTSD. J Psychoactive Drugs.

[32] Feduccia AA, Jerome L, Yazar-Klosinski B, Emerson A, Mithoefer MC, Doblin R (2019). Breakthrough for trauma treatment: safety and efficacy of MDMA-assisted psychotherapy compared to paroxetine and sertraline. Front Psychiatry.

[33] Bershad AK, Mayo LM, Van Hedger K, McGlone F, Walker SC, de Wit H (2019). Effects of MDMA on attention to positive social cues and pleasantness of affective touch. Neuropsychopharmacology.

[34] Corey VR, Pisano VD, Halpern JH (2016). Effects of 3,4-methylenedioxymethamphetamine on patient utterances in a psychotherapeutic setting. J Nerv Ment Dis.

[35] Gurel NZ, Wittbrodt MT, Jung H (2020). Transcutaneous cervical vagal nerve stimulation reduces sympathetic responses to stress in posttraumatic stress disorder: a double-blind, randomized, sham controlled trial. Neurobiol Stress.

[36] Bremner JD, Gurel NZ, Jiao Y (2020). Transcutaneous vagal nerve stimulation blocks stress-induced activation of Interleukin-6 and interferon-γ in posttraumatic stress disorder: a double-blind, randomized, sham-controlled trial. Brain Behav Immun.

[37] Lee DJ, Schnitzlein CW, Wolf JP, Vythilingam M, Rasmusson AM, Hoge CW (2016). Psychotherapy versus pharmacotherapy for posttraumatic stress disorder: systemic review and meta-analyses to determine first-line treatments. Depress Anxiety.

[38] Lamb DG, Porges EC, Lewis GF, Williamson JB (2017). Non-invasive vagal nerve stimulation effects on hyperarousal and autonomic state in patients with posttraumatic stress disorder and history of mild traumatic brain injury: preliminary evidence. Front Med (Lausanne).

[39] Geracioti TD Jr, Baker DG, Ekhator NN (2001). CSF norepinephrine concentrations in posttraumatic stress disorder. Am J Psychiatry.

[40] Young MB, Andero R, Ressler KJ, Howell LL (2015). 3,4-methylenedioxymethamphetamine facilitates fear extinction learning. Transl Psychiatry.

[41] Esber GR, Roesch MR, Bali S (2012). Attention-related Pearce-Kaye-Hall signals in basolateral amygdala require the midbrain dopaminergic system. Biol Psychiatry.

[42] Koenigs M, Huey ED, Raymont V, Cheon B, Solomon J, Wassermann EM, Grafman J (2008). Focal brain damage protects against post-traumatic stress disorder in combat veterans. Nat Neurosci.

[43] Carhart-Harris RL, Murphy K, Leech R (2015). The effects of acutely administered 3,4-methylenedioxymethamphetamine on spontaneous brain function in healthy volunteers measured with arterial spin labeling and blood oxygen level-dependent resting state functional connectivity. Biol Psychiatry.

[44] Vollenweider FX, Gamma A, Liechti M, Huber T (1998). Psychological and cardiovascular effects and short-term sequelae of MDMA ("ecstasy") in MDMA-naïve healthy volunteers. Neuropsychopharmacology.

